# Long non-coding RNA *PXN-AS1* promotes glutamine synthetase-mediated chronic myeloid leukemia BCR::ABL1-independent resistance to Imatinib via cell cycle signaling pathway

**DOI:** 10.1186/s12935-024-03363-9

**Published:** 2024-05-29

**Authors:** Yifei Li, Shiyi Yuan, Ying Zhou, Jingwen Zhou, Xuan Zhang, Ping Zhang, Wenrui Xiao, Ying Zhang, Jianchuan Deng, Shifeng Lou

**Affiliations:** 1 Department of Hematology, The Second Affiliated Hospital of Chongqing Medical University, Chongqing Medical University, Chongqing, 400010 China; 2 Department of Oncology, Southwest Hospital, Army Medical University (Third Military Medical University), Chongqing, 400316 China

**Keywords:** CML, GS, *PXN-AS1*, BCR::ABL1-independent resistance, Cell cycle

## Abstract

**Background:**

Chronic myeloid leukemia (CML) is a common hematological malignancy, and tyrosine kinase inhibitors (TKIs) represent the primary therapeutic approach for CML. Activation of metabolism signaling pathway has been connected with BCR::ABL1-independent TKIs resistance in CML cells. However, the specific mechanism by which metabolism signaling mediates this drug resistance remains unclear. Here, we identified one relationship between glutamine synthetase (GS) and BCR::ABL1-independent Imatinib resistance in CML cells.

**Methods:**

GS and *PXN-AS1* in bone marrow samples of CML patients with Imatinib resistance (IR) were screened and detected by whole transcriptome sequencing. GS expression was upregulated using LVs and blocked using shRNAs respectively, then GS expression, Gln content, and cell cycle progression were respectively tested. The CML IR mice model were established by tail vein injection, prognosis of CML IR mice model were evaluated by Kaplan–Meier analysis, the ratio of spleen/body weight, HE staining, and IHC. *PXN-AS1* level was blocked using shRNAs, and the effects of *PXN-AS1* on CML IR cells in vitro and in vivo were tested the same as GS. Several RNA-RNA tools were used to predict the potential target microRNAs binding to both GS and *PXN-AS1*. RNA mimics and RNA inhibitors were used to explore the mechanism through which *PXN-AS1* regulates *miR-635 *or* miR-635* regulates GS.

**Results:**

GS was highly expressed in the bone marrow samples of CML patients with Imatinib resistance. In addition, the lncRNA *PXN-AS1* was found to mediate GS expression and disorder cell cycle in CML IR cells via mTOR signaling pathway. *PXN-AS1* regulated GS expression by binding to *miR-635*. Additionally, knockdown of *PXN-AS1* attenuated BCR::ABL1-independent Imatinib resistance in CML cells via *PXN-AS1*/*miR-635*/GS/Gln/mTOR signaling pathway.

**Conclusions:**

Thus, *PXN-AS1* promotes GS-mediated BCR::ABL1-independent Imatinib resistance in CML cells via cell cycle signaling pathway.

**Graphic Abstract:**

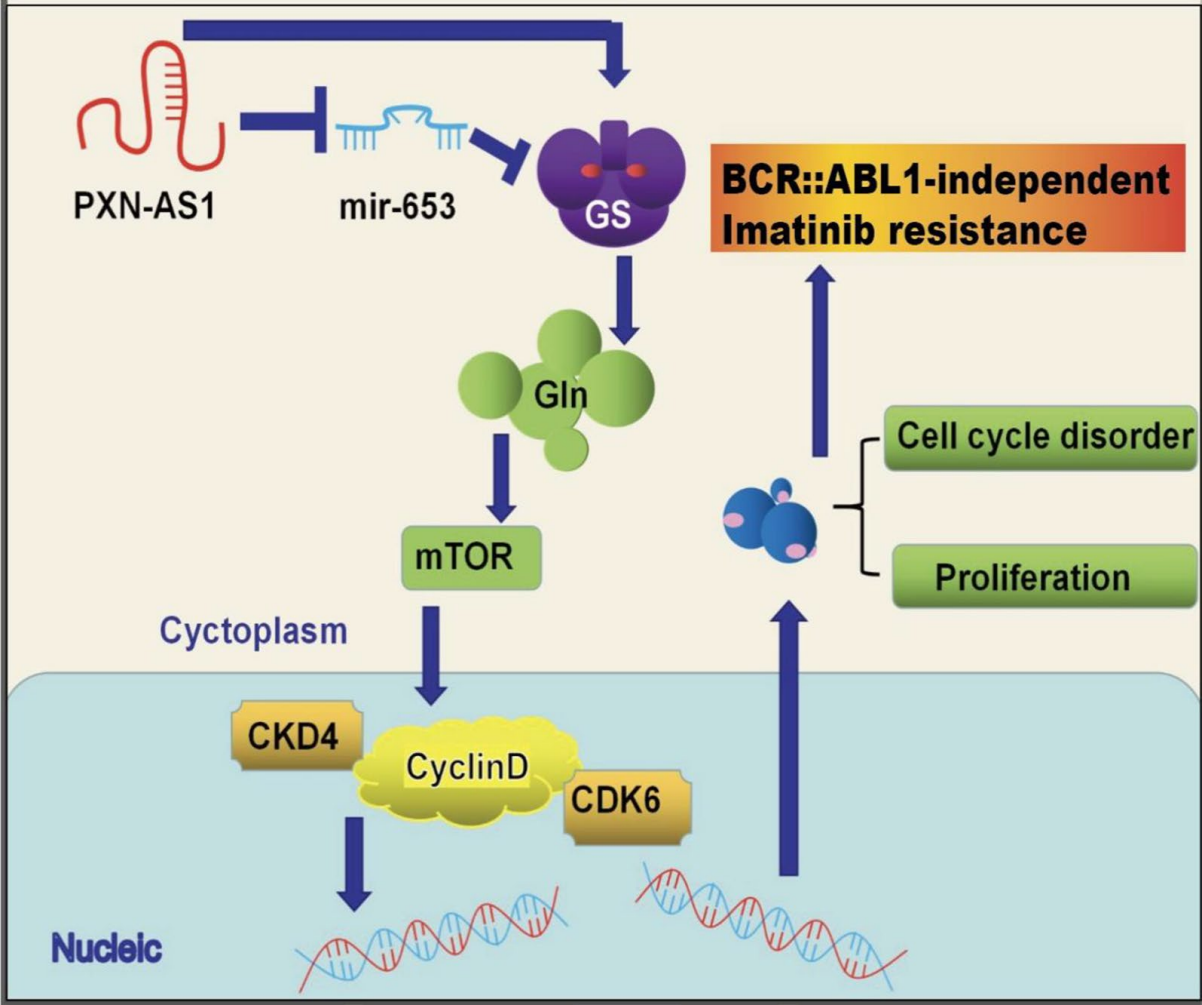

**Supplementary Information:**

The online version contains supplementary material available at 10.1186/s12935-024-03363-9.

## Background

Chronic myeloid leukemia (CML) is a common hematopoietic malignancy all over world, which is caused by a fusion gene known as BCR::ABL1, one type of tyrosine kinase [1–4]. As for the molecular mechanism of drug resistance in CML cells, there are several primary types: BCR::ABL1 mutations, BCR::ABL1-independent resistance, abnormal intracellular transporter of Imatinib, and survival of CML stem cells [5–10]. Some scholars have proposed that BCR::ABL1-independent resistance is the most common cause of drug resistance in CML patients [11]. Recent studies demonstrated that PI3K/AKT/mTOR signaling pathway of CML cells might play an imperative role in BCR::ABL1-independent drug resistance [12, 13]. However, there is limited research on BCR::ABL1-independent Imatinib resistance. Therefore, it is imperative and time-critical to delve into the underlying mechanisms of BCR::ABL1-independent drug resistance in CML patients, which will enable the development of innovate and effective therapeutic strategies to benefit CML patients.

Dysregulation of cellular metabolism is a significant hallmark of cancer, which has something to do with the initiation and progression of tumors [14, 15]. Glutamine (Gln), an essential amino acid in human metabolism, is involved in various biological processes. As one of symbolic characteristics of cancer, activated cellular metabolism involves in both tumorigenesis and tumor progression [16, 17]. Glutamine synthetase (GS), encoded by the gene *GLUL*, is an enzyme that catalyzes glutamate and ammonium ions into Gln, which can be further broken down by glutaminase in the body [18]. In previous studies, targeting stromal GS in an ovarian cancer model has been shown to induce tumor regression [19]. Another study demonstrated that targeting the GS-mTORC1 axis in liver cancer can improve precision medicine therapy [20]. Additionally, our previous study found that targeting GS could enhance the chemosensitivity of colon cancer cells to 5-fluorouracil, a common chemotherapy drug [21]. Long non-coding RNAs (lncRNAs) have been found to exhibit abnormal expression patterns in various types of cancers [22, 23]. They can serve as diagnostic basis or potential therapeutic targets of cancer. PXN antisense transcript 1 (*PXN-AS1*) is a specific type of lncRNA that has been reported to be involved in tumorigenesis. It is associated with various aspects of cancer development and progression. PXN-AS1 has been found to play a promoting role in the malignancy of several types of cancer, including hepatocellular carcinoma [24, 25], non-small lung cancer [26], nasopharyngeal carcinoma [27], and glioblastoma [28, 29]. In addition, lncRNA often forms the lncRNA/miRNA/mRNA regulatory axis together with miRNA and mRNA, which is involved in the regulation of various tumor-related signaling pathways, thereby regulating different pathological processes such as tumor cell invasion, metastasis, immune escape and drug resistance [30, 32]. However, the specific function of *PXN-AS1* in CML is currently unknown and requires further investigation.

Herein, we reported that the lncRNA *PXN-AS1* could promote BCR::ABL1-independent resistance to Imatinib in CML by upregulating GS expression. Our results demonstrated that GS was highly expressed in CML cell lines and peripheral blood samples of CML patients. Moreover, we found that the elevated GS levels were positively associated with BCR::ABL1-independent resistance to Imatinib in CML via activation of the Cyclin D-CDK4/6 complex. In addition, lncRNA *PXN-AS1* promoted BCR::ABL1-independent resistance to Imatinib in CML through the *PXN-AS1*/*miR-635*/GS/Gln/mTOR/Cyclin D-CDK4/6 complex signaling pathway. This novel regulatory axis involving *PXN-AS1*, *miR-635*, GS, mTOR, and Cyclin D-CDK4/6 contributes to the development of drug resistance in CML patients. Therefore, targeting the *PXN-AS1*/*miR-635*/GS/Gln/mTOR axis might serve as a potential therapeutic target for overcoming Imatinib resistance in CML patients.

## Methods

### Cell lines and culture

The human chronic leukemia cell lines K562 and LAMA84, as well as human renal epithelial cell line HEK-293T were purchased from American Type Culture Collection (ATCC; Manassas, VA, USA). K562 and LAMA84 cells were induced into Imatinib-resistant (IR) CML cells by our group. Imatinib was dissolved in DMSO for CML IR cells cultivation. Moreover, MSO was also dissolved in DMSO for further use. K562 IR cells were cultured in RPMI 1640 medium (Gibco, Grand Island, NY, USA) supplemented with 10% fetal bovine serum (FBS), 1% penicillin–streptomycin (Beyotime, Shanghai, China), and 200 nM Imatinib (Haosen, Jiangsu, China). LAMA84 IR cells were cultured in RPMI 1640 medium supplemented with 10% FBS (Gibco), 1% penicillin–streptomycin and 100 nM Imatinib. HEK-293T cells were cultured in DMEM (Gibco) supplemented with 10% FBS (Gibco) and, 1% penicillin–streptomycin. All cells were maintained at 37 °C in a humidified atmosphere with 5% CO_2_. For Gln deprivation, K562 IR cells and LAMA84 IR cells were cultivated overnight in complete RPMI 1640 medium. After washing with PBS, the cells were transferred into Gln-free RPMI 1640 medium supplemented with 2% FBS. The CML IR cells were either Gln-starved or cultivated with 4 mM Gln (ThermoFisher Scientific, USA) for another 24 h prior to further analysis.

### Bone marrow samples of CML patients

Bone marrow samples of CML patients were collected from the Second Affiliated Hospital Chongqing Medical University in 2021. Ethical approval for the use of human subjects was permitted by the Research Ethics Committee of the Second Affiliated Hospital of Chongqing Medical University. The CML patients were divided into two groups, one is new diagnostic group, the other is primary resistant group. Both group members were absent of BCR::ABL1 mutation. Leukocytes were isolated from the bone marrow samples of CML patients using the Human Blood Mononuclear Cell Isolation Kit (Tianjin Haoyang Biological Manufacture Co., Ltd., Tianjin, China). The leukocytes were then cultured in complete RPMI 1640 medium supplemented with 10% FBS before RNA isolation or protein isolation. Detailed information of patients is shown in Additional file 1: Table S7.

### Whole transcriptome sequencing

Peripheral blood leukocytes isolated from the bone marrow samples of CML patients was treated with TRIzol reagent (Beyotime, Shanghai, China) for total RNA harvest. The RNA amount and purity of each sample was quantified by NanoDrop ND-1000 (NanoDrop, Wilmington, DE, USA). Then the cleaved RNA fragments were reverse-transcribed to create the cDNA by SuperScript™ II Reverse Transcriptase (Invitrogen, CA, USA). Then the ligated products are amplified with PCR, the average insert size for the final cDNA library was 300 ± 50 bp. At last, the 2 × 150 bp paired-end sequencing (PE150) was performed on an illumina Novaseq™ 6000 (LC-Bio Technology CO., Ltd., Hangzhou, China) following the vendor's recommended protocol. FASTP were used to remove the reads that contained adaptor contamination, low quality bases and undetermined bases with default parameter. Then sequence quality was also verified by FASTP. Genes differential expression analysis was performed by DESeq2 software between two different groups (and by edgeR between two samples). The genes with the parameter of false discovery rate (FDR) below 0.05 and absolute fold change ≥ 2 were considered differentially expressed genes. Differentially expressed genes were then subjected to enrichment analysis of GO functions and KEGG path. Whole transcriptome sequencing of peripheral blood leukocytes was conducted and analyzed by Lianchuan Biotechnology Co., Ltd..

### Real-time PCR analysis

Total RNA was isolated from the peripheral blood leukocytes of CML patients and CML IR cell lines by use of TRIzol reagent (Beyotime). Reverse transcription of the extracted RNA to cDNA was done by using the PrimeScript RT Reagent Kit with gDNA Eraser (Takara, Shiga, Japan). Real-time PCR was conducted by using the SYBR Premix Ex Taq (Takara). The primer sequences are shown in Additional file 1: Table S1 and Table S2. Finally, the relative mRNA expression of each target gene was determined.

### Western blotting

Total protein of the leukocytes of CML patients and CML IR cell lines were extracted and isolated through SDS–polyacrylamide gel electrophoresis (Beyotime), and transferred onto a polyvinylidene fluoride membrane (Millipore, Burlington, MA, USA). The following primary antibodies were used for the assay: anti-GS (Affinity, Jiangsu, China), anti-mTOR (Affinity), anti-Cyclin D1 (Affinity), anti-CDK4 (Affinity), anti-CDK6 (Affinity), anti-PCNA (Affinity), anti-Tubulin (Proteintech, Rosemont, IL, USA), anti-GAPDH (Proteintech), and anti-β-actin (Proteintech).

### Lentiviral infection

Human LV4-GS, LV4-NC, sh-GS, sh-*PXN-AS1*, and sh-NC were designed by Tsingke Biotechnology Co., Ltd. (Beijing, China). K562 IR cells and LAMA84 IR cells were transfected with Lipofectamine 2000 (Invitrogen, Waltham, MA, USA). Related nucleic acid sequences are shown in Additional file 1: Table S3.

### Transient transfection

Human *miR-635* mimic, NC mimic, *miR-635* inhibitor, and NC inhibitor were designed by Tsingke Biotechnology Co., Ltd. (Beijing, China). K562 IR cells and LAMA84 IR cells were transfected using Lipofectamine 2000 (Invitrogen, Waltham, MA, USA). Related nucleic acid sequences are shown in Additional file 1: Table S4 and Table S5.

### Measurement of Gln content

Gln contents of peripheral blood leukocytes of CML patients and CML IR cell lines were measured by use of a Gln Content Measuring Kit (Solarbio, Beijing, China) in accordance with the manufacturer’s protocol. The OD value of treated CML IR cells at A450 were tested by using a FACS Calibur flow cytometer (BD Company, USA). Then, the Gln content was calculated according to the manufacturer’s protocol.

### Cell Counting Kit-8 assays

Cell proliferation was examined using the Cell Counting Kit-8 (CCK-8; Dojindo Molecular Technologies, Kumamoto, Japan). After transfection, the cells were diluted at a density of 2 × 10^3^ cells per well and then incubated in 96-well culture plates. The OD value of each well was tested and culture medium was changed every 24 h until 72 h.

### Cell cycle analysis

After being fixed with 70% ethyl alcohol at 4 °C for 10 h, the CML IR cells were incubated in 50 μL RNase (Beyotime) at room temperature for 30 min. Nextly, 400 μL of propidium iodide (Beyotime) was added to each tubes, then incubated at room temperature for another 30 min. Finally, the stained CML IR cells were tested by using a FACS Calibur flow cytometer (BD Company, USA).

### Dual-luciferase reporter assays

Then HEK-293T cells were co-transfected with either wild-type (WT) GS luciferase reporter vectors or mutant (Mut) GS luciferase reporter vectors and either *miR-635* mimic or NC mimic. At the same time, HEK-293T cells were co-transfected with either WT *PXN-AS1* luciferase reporter vectors or Mut *PXN-AS1* luciferase reporter vectors and either *miR-635* mimic or NC mimic. After co-transfection for two days, the luciferase activity of each well was tested by using multimode reader.

### Animal studies

Half male and half female BALB/c nude mice (4–6 weeks old) were obtained from Weitonglihua Experimental Animal Technology Co., Ltd. (Beijing, China) and raised under standard conditions in accordance with the institutional guidelines for animal care in experimental animal center of Chongqing Medical University. Animal study was approved by Laboratory Animal Management and Use Committee of the Second Affiliated Hospital of Chongqing Medical University. To establish an CML IR mouse model, 1 × 10^7^ cells/L of cells were injected into the BABL/c nude mice via tail vein. All mice were re-injected with the same cell concentration a week later. Each group received different treatments after injection based on the group information. After 4–5 weeks, the mice were sacrificed for their spleens. After fixing with formaldehyde for 1 week, the sections were embedded in paraffin before HE staining and IHC.

### Hematoxylin and Eosin (HE) staining

The spleen paraffin sections were deparaffinized in graded xylene and ethanol, followed by staining with hematoxylin (Beyotime) for 30 min and eosin (Beyotime) for 3 min. Subsequently, the sections were dehydrated using a series of graded ethanol solutions and xylene. After dehydration, a coverslip was applied using resinene (Solarbio).

### Immunohistochemistry (IHC)

First, the spleen paraffin sections were deparaffinized in xylene and graded ethanol. To block endogenous peroxidase activity, the sections were incubated with 10% hydrogen peroxide. For antigen retrieval, the sections were heated in 2% EDTA solution for 15 min, and allowed to cool for 2 h at room temperature. After washing with PBS, the slides were blocked with goat serum (Zhongshanjinqiao Biotechnology Co., Ltd., China) for 15 min, and then incubated with the diluted antibodies at 4℃ overnight. On the next day, the sections were incubated with corresponding secondary antibodies at room temperature for 15 min. The sections were then labeled with horseradish peroxidase, and the signals were detected using the DAB Peroxidase Substrate Kit (Solarbio). After staining with hematoxylin (Beyotime) for 30 s, the slides were dehydrated in graded ethanol and xylene. Finally, the sections were sealed with coverslips by resinene (Solarbio). The following primary antibodies were used for the assay: anti-GS (Affinity), anti-mTOR (Affinity), anti-Cyclin D1 (Affinity), anti-CDK4 (Affinity), anti-CDK6 (Affinity).

### Bioinformatics tools

Four online tools (Targetscan, Tarbase, miRDB, and miRWalk) for RNA-RNA interaction analysis were available for free, which were used for predicting the potential binding sites. Information of the four tools is shown in Additional file 1: Table S6.

### Statistical analysis

Statistical analyses were operated with SPSS PASW (version 24.0; IBM, Armonk, NY, USA), and the diagrams were drawn by using GraphPad Prism (version 9.0; GraphPad Software Inc., San Diego, CA, USA). All values are expressed as mean ± standard deviation. The levels of statistical significance were set at *P < 0.05, **P < 0.01, and ***P < 0.005; while "NS" was used to denote results that were not statistically significant.

## Results

### GS expression is increased in the bone marrow of CML patients resistant to Imatinib and in CML IR cell lines

**Fig. 1:**
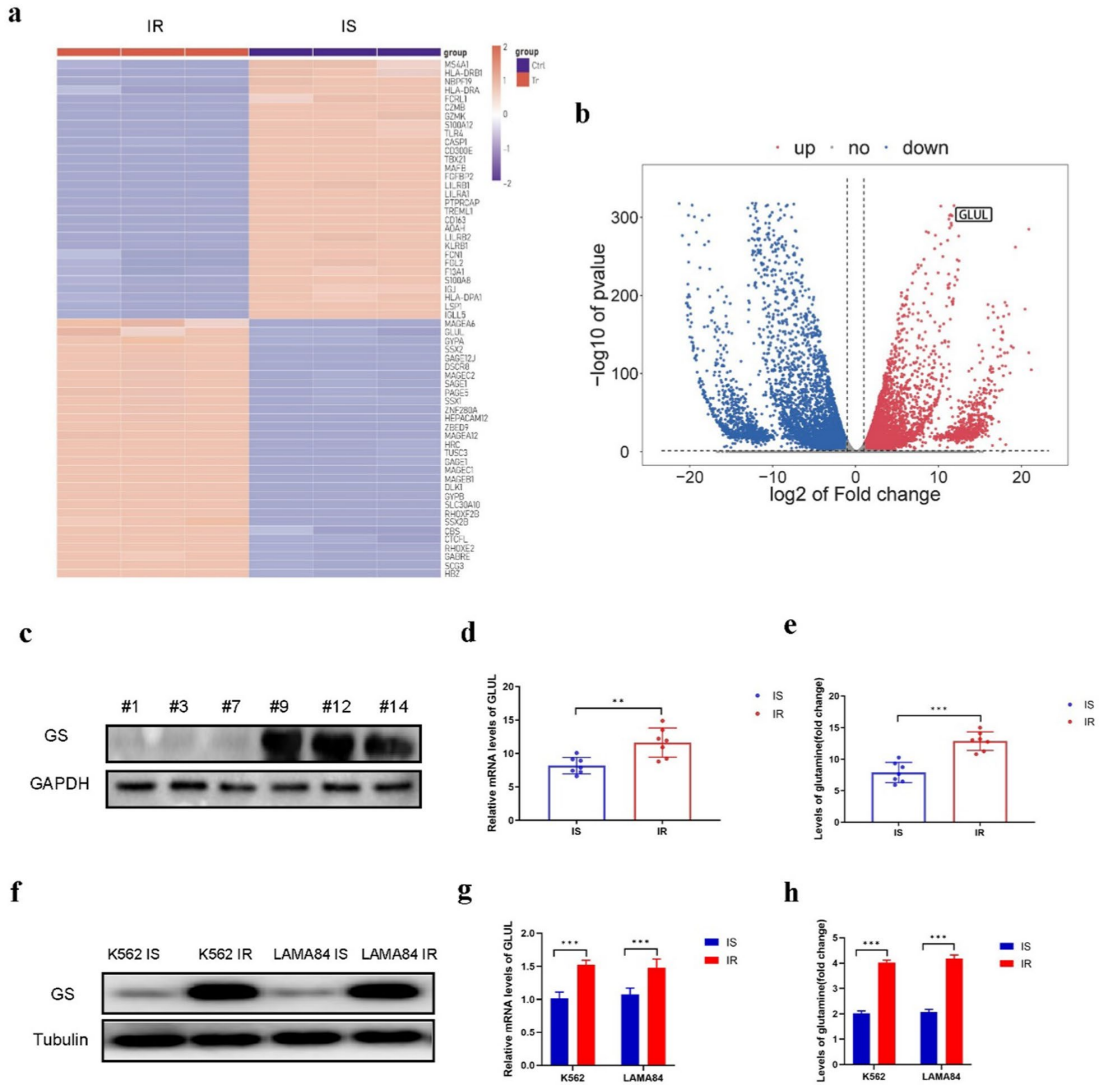
Increased GS expression in bone marrow of CML patients resistant to Imatinib and CML Imatinib resistance cell lines. **a** Heatmap of target genes between bone marrow of CML patients resistant to Imatinib and those sensitive to Imatinib screened by RNA-sequencing. **b** volcano plot of target genes between bone marrow of CML patients sensitive to Imatinib vs those resistant to Imatinib screened by RNA-sequencing. **c** Western blot of GS expression in bone marrow of CML patients resistant to Imatinib (#9,#12,#14) and those sensitive to Imatinib (#1,#3,#7). **d** Real-time PCR of GS expression in bone marrow of CML patients resistant to Imatinib and those sensitive to Imatinib. **e** Gln content in bone marrow of CML patients resistant to Imatinib and those sensitive to Imatinib by Gln content assay kit. **f** Western blot of GS expression between two CML cell lines resistant to Imatinib and those sensitive to Imatinib. **g** Real-time PCR of GS expression between two CML cell lines resistant to Imatinib and those sensitive to Imatinib. **h** Gln content between two CML cell lines resistant to Imatinib and those sensitive to Imatinib by Gln content assay kit. Data are expressed as mean ± standard deviation of at least three independent experiments. Statistical significance was concluded at *P < 0.05, **P < 0.01, and ***P < 0.005

GS is the limiting enzyme involving in the process of Gln biosynthesis and is vital for tumorigenesis as well as cancer progression. To investigate the significance of GS in CML resistance to Imatinib, we initially conducted RNA-seq analysis on three pairs of bone marrow samples from CML patients who showed resistance or sensitivity to Imatinib. This analysis allowed us to identify and categorize the upregulated genes and pathways. Numerous genes, such as *GLUL*, were determined to be highly enriched in the samples of CML patients resistant to Imatinib (Fig. 1a, b). Additionally, we observed activation of cell cycle signaling pathways during our analysis (Fig. S1a-d). Next, we conducted separate tests to determine the level of GS expression and the content of Gln in the bone marrow samples of CML patients who were resistant or sensitive to Imatinib. The protein and mRNA expression levels of GS were highly increased in CML patients resistant to Imatinib than those sensitive to Imatinib, as revealed by Western blotting and RT-PCR assays respectively (Fig. 1c, d). The levels of Gln in the samples of CML patients who were resistant to Imatinib compared to those who were sensitive to Imatinib were evaluated using the Gln Content Assay Kit (Fig. 1e). Next, two human CML cell lines, K562 and LAMA84, were chosen for further experimentation. These cell lines were subjected to a gradual increase in the dosage of Imatinib to induce resistance. The resulting Imatinib-resistant cell lines were designated as K562 IR and LAMA84 IR respectively. Western blot analysis revealed that GS expression was highly increased in K562 IR cells and LAMA84 IR cells (Fig. 1f). Similarly, RT-PCR assays indicated the mRNA level of GS was increased in K562 IR cells and LAMA84 IR cells (Fig. 1g). Furthermore, using the Gln Content Assay Kit, we observed an elevation in the levels of Gln in K562 IR cells and LAMA84 IR cells (Fig. 1h). These data demonstrated that GS was upregulated in the human bone marrow of CML patients who were Imatinib resistant and human Imatinib resistant CML cell lines, implying that GS might be a key factor for Imatinib resistance in CML patients.

### Overexpression of GS promotes CML cell resistant to Imatinib by perturbing cell cycle of CML cells via mTOR signaling pathway both in vitro and in vivo

**Fig. 2:**
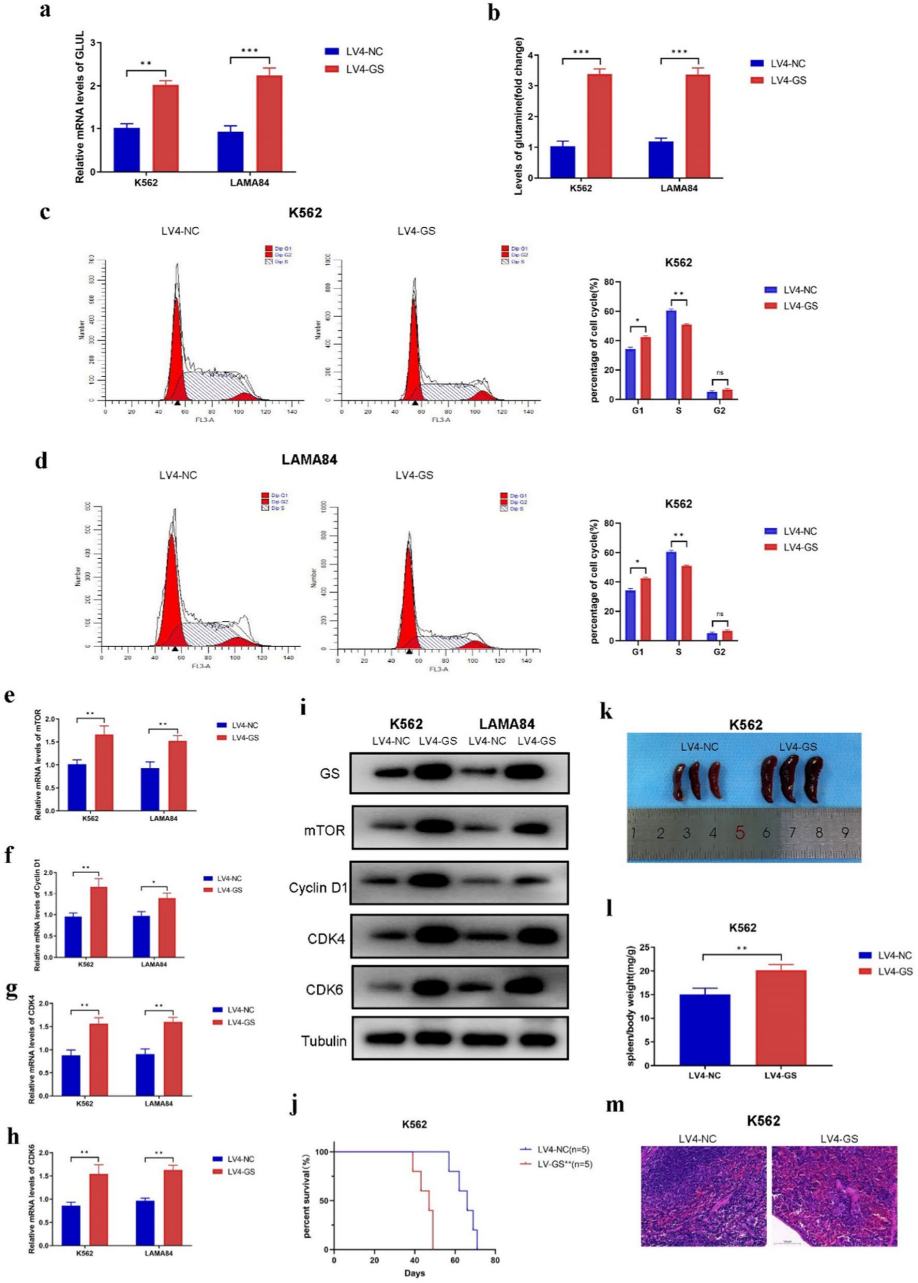
GS overexpression perturbed cell cycle of CML cells in vitro and in vivo.** a** Real-time PCR of GS expression in LV4-GS-transfected K562 cells and LAMA84 cells. **b** Gln content of LV4-GS-transfected K562 cells and LAMA84 cells. **c** Cell cycle percentage of K562 cells after LV4-GS transfection. **d** Cell cycle percentage of LAMA84 cells after LV4-GS transfection. **e** Real-time PCR of mTOR expression in LV4-GS-transfected K562 cells and LAMA84 cells. **f** Real-time PCR of Cyclin D1 expression in LV4-GS-transfected K562 cells and LAMA84 cells. **g** Real-time PCR of CDK4 expression in LV4-GS-transfected K562 cells and LAMA84 cells. **h** Real-time PCR of CDK6 expression in LV4-GS-transfected K562 cells and LAMA84 cells. **i** Western blot of GS, mTOR, Cyclin D1, CDK4, and CDK6 expression in LV4-GS-transfected K562 cells and LAMA84 cells. **j** Kaplan–Meier analysis of K562^LV4-GS^-injected mice. **k** Overall spleen size of K562^LV4-GS^-injected mice. **l** The ratio of spleen/body weight of K562^LV4-GS^-injected mice. **m** Representative images of HE staining of spleen in K562^LV4-GS^-injected mice. Data are expressed as mean ± standard deviation of at least three independent experiments. Statistical significance was concluded at *P < 0.05, **P < 0.01, and ***P < 0.005

To explore the effects of GS on Imatinib resistant CML cells, both lentivirus and shRNA were used respectively. First, lentivirus was used to upregulate the expression of GS in K562 and LAMA84 cells, which were both sensitive to Imatinib. RT-PCR analysis revealed that the mRNA levels of GS were increased in K562^LV4-GS^ and LAMA84^LV4-GS^ cells compared to K562^LV4-NC^ and LAMA84^LV4-NC^ cells (Fig. 2a). According to the results of Gln Content Assay, the content of Gln was also increased in K562^LV4-GS^ and LAMA84^LV4-GS^ cells compared to K562^LV4-NC^ and LAMA84^LV4-GS^ cells (Fig. 2b). In the context of the proliferation of K562^LV4-GS^ and LAMA84^LV4-GS^ cells, it was observed that the mRNA levels of PCNA were increased in these cells (Fig. S2a). According to the results of CCK-8 assays, GS overexpression promoted the ability of proliferation of K562^LV4-GS^ and LAMA84^LV4-GS^ cells (Fig. S2b, c). Additionally, the cell cycle progression of LV4-GS-transfected CML cells were evaluated by flow cytometry. The results showed that GS overexpression could prolong the G1-phase of K562^LV4-GS^ and LAMA84^LV4-GS^ cells (Fig. 2c, d). The relative mRNA levels of mTOR, Cyclin D1, CDK4, and CDK6 were also increased in LV4-GS-transfected CML cells (Fig. 2e–h). Moreover, the expression levels of mTOR, Cyclin D1, CDK4, and CDK6 were also upregulated in K562^LV4-GS^ and LAMA84^LV4-GS^ cells (Fig. 2i). These data suggested that GS could promote the proliferation of CML cells and disordered the cell cycle of CML cells via mTOR signaling pathway in vitro.

To determine the function of GS on CML in vivo, a CML mouse model was established by tail vein injection. Based on the results of the Kaplan–Meier analysis, overexpression of GS was found to be a predictor of poor prognosis (Fig. 2j). The symptom of splenomegaly was more severe in K562^LV4-GS^-injected mice than in K562^LV4-NC^-injected mice (Fig. 2k). In addition, the ratio of spleen/body weight was significantly higher in K562^LV4-GS^-injected mice (Fig. 2l). HE staining was conducted to detect the presence of spleen metastasis in CML cells. It was found that K562^LV4-GS^-injected mice had more spleen metastasis CML cells than K562^LV4-NC^-injected mice (Fig. 2m). In addition, IHC staining was performed to detect the expression levels of GS, mTOR, Cyclin D1, CDK4, and CDK6. The results indicated that the expression levels of GS, mTOR, Cyclin D1, CDK4, and CDK6 were all increased in the spleen tissues of K562^LV4-GS^-injected mice (Fig. S3). These data demonstrated that overexpression of GS could accelerate the progression of CML and predict a worsen prognosis.

### GS knockdown of CML IR cells alleviates the Imatinib resistance both in vitro and in vivo

**Fig. 3:**
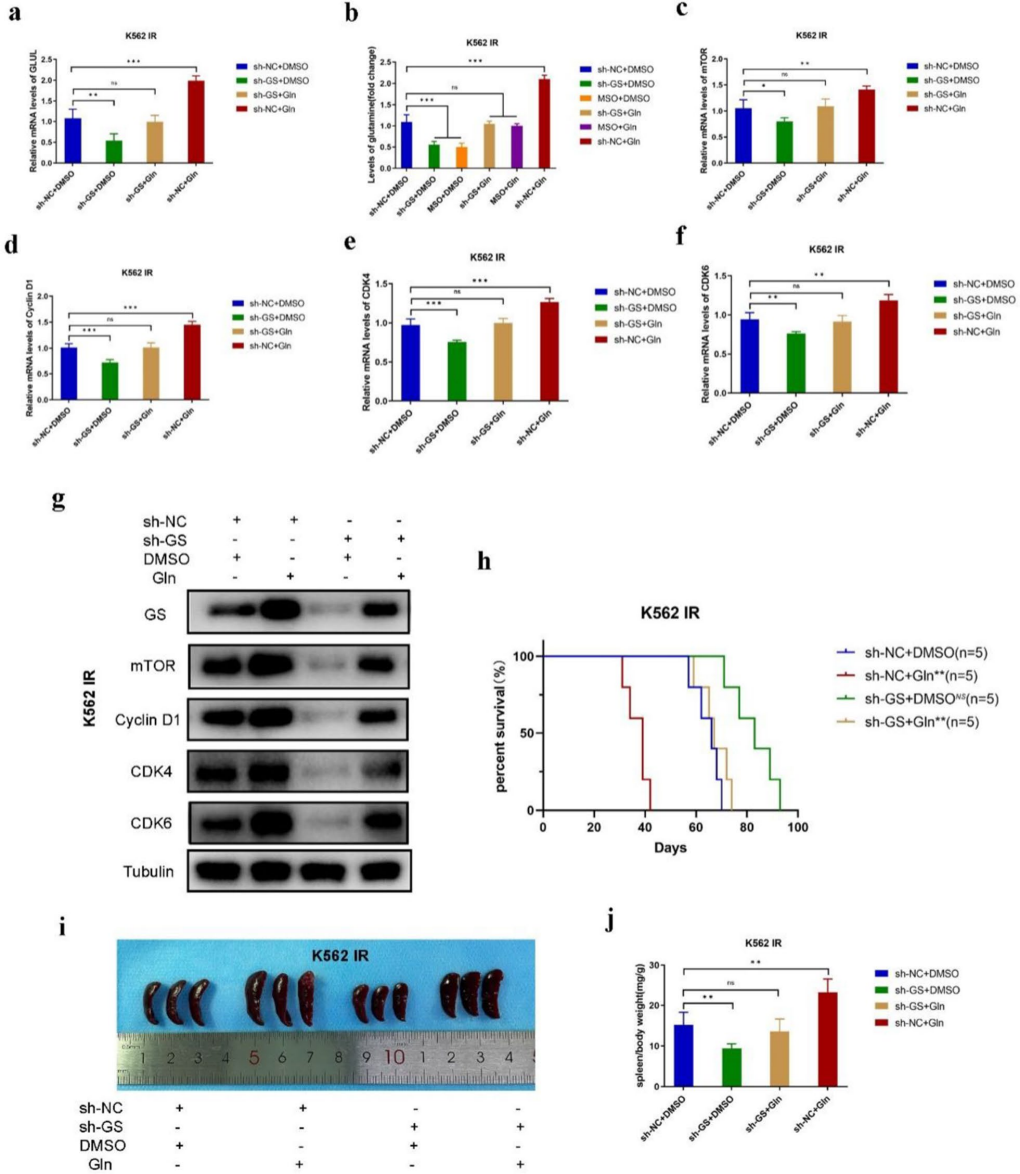
Knockdown GS inhibited mTOR signaling and Cyclin D/CDK4-CDK6 complex in vitro and in vivo. **a** Real-time PCR of GS expression in sh-GS-transfected K562 IR cells. **b** Gln content of sh-GS-transfected K562 IR cells. **c** Real-time PCR of mTOR expression in sh-GS-transfected K562 IR cells. **d** Real-time PCR of Cyclin D1 expression in sh-GS-transfected K562 IR cells. **e** Real-time PCR of CDK4 expression in sh-GS-transfected K562 IR cells. **f** Real-time PCR of CDK6 expression in sh-GS-transfected K562 IR cells. **g** Western blot of GS, mTOR, Cyclin D1, CDK4, and CDK6 expression in sh-GS-transfected K562 IR cells. **h** Kaplan–Meier analysis of K562 IR^sh-GS^-injected mice. **i** Overall spleen size of K562 IR^sh-GS^-injected mice. **j** The ratio of spleen/body weight of K562 IR^sh-GS^-injected mice. Data are expressed as mean ± standard deviation of at least three independent experiments. Statistical significance was concluded at *P < 0.05, **P < 0.01, and ***P < 0.005

To further explore the effect of GS on CML IR cells, shRNA was used to inhibit GS expression. RT-PCR analysis revealed that the relative mRNA levels of GS were decreased in K562 IR^sh-GS^ and LAMA84 IR^sh-GS^ cells compared to K562 IR^sh-NC^ and LAMA84 IR^sh-NC^ cells (Fig. 3a and Fig. S4a). Methionine sulfone (MSO) was the specific inhibitor of GS, which was used for contrasting the inhibition efficiency of shRNA. Moreover, the content of Gln was also decreased in K562 IR^sh-GS^ and LAMA84 IR^sh-GS^ cells (Fig. 3b and Fig. S4b). According to the results of CCK-8 assay, GS knockdown suppressed the proliferation of K562 IR^sh-GS^ and LAMA84 IR^sh-GS^ cells (Fig. S5a, b). Furthermore, the relative mRNA levels of PCNA were decreased in K562 IR^sh-GS^ and LAMA84 IR^sh-GS^ cells (Fig. S5c, d). Next, flow cytometry was performed to examine the cell cycle progression of sh-GS-transfected CML IR cells. The results showed that GS inhibition led to a shortened G1 phase in K562 IR^sh-GS^ and LAMA84 IR^sh-GS^ cells (Fig. S5e, f). The relative mRNA levels of mTOR, Cyclin D1, CDK4, and CDK6 were also decreased in sh-GS-transfected CML IR cells (Fig. 3c–f and Fig. S4c–f). Furthermore, the expression of mTOR, Cyclin D1, CDK4, and CDK6 were also downregulated in K562 IR^sh-GS^ and LAMA84 IR^sh-GS^ cells (Fig. 3g and Fig. S4g). These data demonstrated that GS knockdown could restrain the growth of CML IR cells and rescue the cell cycle disorder of CML IR cells via suppressing mTOR signaling pathway in vitro.

Moreover, the CML mouse model was established via tail vein injection. The Kaplan–Meier analysis revealed that GS inhibition could predict favorable prognosis (Fig. 3h). The symptom of splenomegaly was less severe in K562 IR^sh-GS^-injected mice than in K562 IR^sh-NC^-injected mice (Fig. 3i). In addition, the ratio of spleen/body weight was much lower in K562 IR^sh-GS^-injected mice (Fig. 3j). The results of HE staining showed that K562 IR^sh-GS^-injected mice had more spleen metastasis CML cells than K562 IR^LV4-NC^-injected mice (Fig. S6a). Next, IHC staining was performed to explore the expression levels of GS, mTOR, Cyclin D1, CDK4, and CDK6. The results indicated that the expression levels of GS, mTOR, Cyclin D1, CDK4, and CDK6 were all decreased in the spleen tissues of K562 IR^sh-GS^-injected mice (Fig. S6b). These data demonstrated that GS knockdown could inhibit the progression of CML and predict a favorable prognosis.

### *PXN-AS1* is increased in the bone marrow samples of CML patients with Imatinib resistance and in CML IR cell lines

**Fig. 4:**
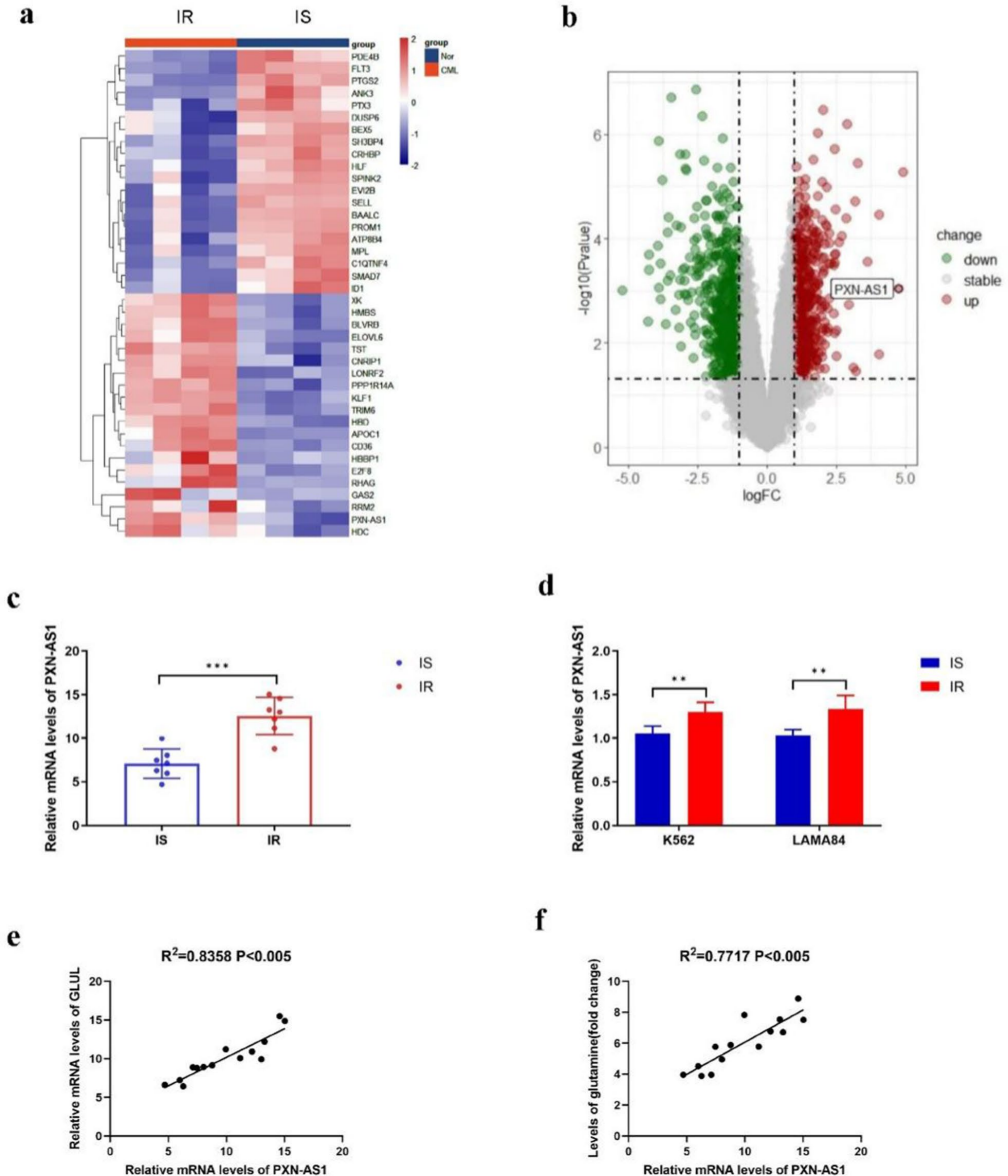
Increased level of *PXN-AS1* in bone marrow of CML patients resistant to Imatinib and CML IR cell lines. **a** Heatmap of target lncRNAs between bone marrow of CML patients resistant to Imatinib and those sensitive to Imatinib screened by RNA-sequencing. **b** Volcano plot of target lncRNAs between bone marrow of CML patients resistant to Imatinib and those sensitive to Imatinib screened by RNA-sequencing. **c** Real-time PCR of relative mRNA levels of *PXN-AS1* in bone marrow of CML patients resistant to Imatinib and those sensitive to Imatinib. **d** Real-time PCR of relative mRNA levels of *PXN-AS1* between two CML cell lines resistant to Imatinib and those sensitive to Imatinib. **e** and** f** Correlation analyses between relative mRNA levels of *PXN-AS1* and GS or Gln levels in 7pairs of bone marrow of CML patients resistant to Imatinib and those sensitive to Imatinib. Data are expressed as mean ± standard deviation of at least three independent experiments. Statistical significance was concluded at *P < 0.05, **P < 0.01, and ***P < 0.005

*PXN-AS1* is classified as a lncRNA that has been implicated in various types of cancer. As a result of the above-mentioned RNA-seq analysis, it was observed that *PXN-AS1* was upregulated in the samples of CML patients who were resistant to Imatinib (Fig. 4a, b). In addition to the upregulation of *PXN-AS1*, the analysis also revealed the activation of pathways related to transcriptional misregulation in cancer signaling. (Fig. S7a, b). By employing RT-PCR analysis, it was observed that the level of *PXN-AS1* was significantly higher in samples from CML patients who were resistant to Imatinib compared to those who were sensitive to Imatinib. (Fig. 4c). Moreover, the levels of *PXN-AS1* were increased in K562 IR and LAMA84 IR cells, as revealed by RT-PCR analysis RT-PCR (Fig. 4d). In addition, *PXN-AS1* expression had positive correlation with GS and Gln levels in CML patients who were resistant to Imatinib (Fig. 4e, f). These data indicated that *PXN-AS1* was upregulated in human bone marrow of CML patients with Imatinib resistance and CML IR cell lines, implying that *PXN-AS1* might serve as a crucial factor for GS-mediated BCR::ABL1-independent Imatinib resistance in CML cells.

### *PXN-AS1* knockdown reverses the cell cycle disorder of CML IR cells both in vitro and in vivo

**Fig. 5:**
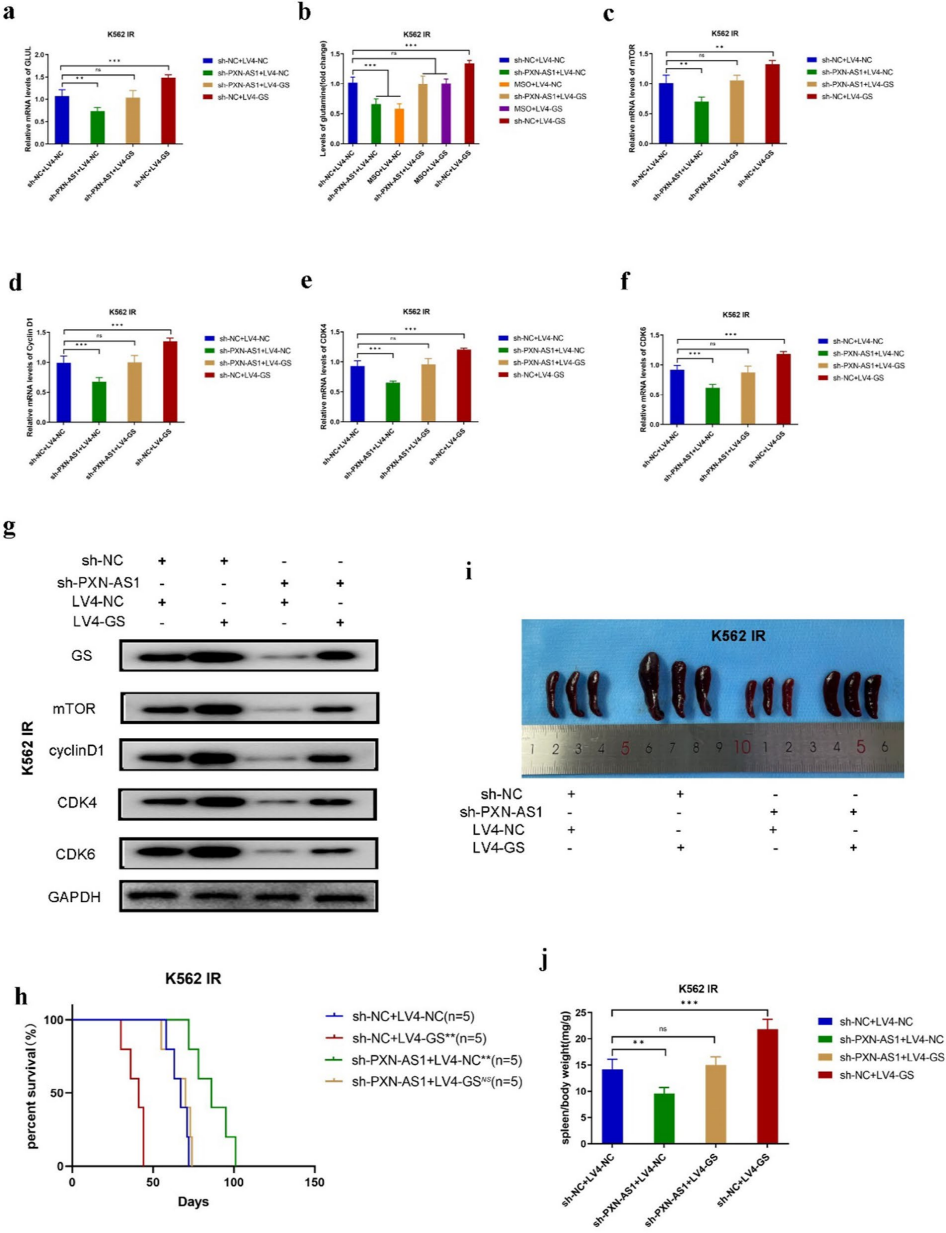
Knockdown *PXN-AS1* inhibited mTOR signaling and Cyclin D/CDK4-CDK6 complex in vitro and in vivo. **a** Real-time PCR of *PXN-AS1* expression in sh-*PXN-AS1*-transfected K562 IR cells. **b** Gln content of sh-*PXN-AS1*-transfected K562 IR cells. **c** Real-time PCR of mTOR expression in sh-*PXN-AS1*-transfected K562 IR cells. **d** Real-time PCR of Cyclin D1 expression in sh-*PXN-AS1*-transfected K562 IR cells. **e** Real-time PCR of CDK4 expression in sh-*PXN-AS1*-transfected K562 IR cells. **f** Real-time PCR of CDK6 expression in sh-*PXN-AS1*-transfected K562 IR cells. **g** Western blot of GS, mTOR, Cyclin D1, CDK4, and CDK6 expression in sh-*PXN-AS1*-transfected K562 IR cells. **h** Kaplan–Meier analysis of K562 IR^sh-*PXN-AS1*^-injected mice. **i** Overall spleen size of K562 IR^sh-*PXN-AS1*^-injected mice. **j** The ratio of spleen/body weight of K562 IR^sh-*PXN-AS1*^-injected mice. Data are expressed as mean ± standard deviation of at least three independent experiments. Statistical significance was concluded at *P < 0.05, **P < 0.01, and ***P < 0.005

To explore the effect of *PXN-AS1* on CML IR cells and the relationship between *PXN-AS1* and GS, shRNA was used for blocking *PXN-AS1* (Fig. S8a, b). RT-PCR assay indicated that relative mRNA levels of GS were significantly decreased in K562 IR^sh-*PXN-AS1*^ and LAMA84 IR^sh-*PXN-AS1*^ cells compared to K562 IR^sh-NC^ and LAMA84 IR^sh-NC^ cells (Fig. 5a and Fig. S10a). Similarly, the content of Gln was also decreased in K562 IR^sh-*PXN-AS1*^ and LAMA84 IR^sh-*PXN-AS1*^ cells (Fig. 5b and Fig. S10b). According to the results of CCK-8 assay, *PXN-AS1* knockdown suppressed the proliferation of K562 IR^sh-*PXN-AS1*^ and LAMA84 IR^sh-*PXN-AS1*^ cells (Fig. S9a, b). In addition, the relative mRNA levels of PCNA were also decreased in K562 IR^sh-*PXN-AS1*^ and LAMA84 IR^sh-*PXN-AS1*^ cells (Fig. S9c,d). Furthermore, cell cycle progression of sh-*PXN-AS1*-transfected CML IR cells were tested by flow cytometry. The results showed that the inhibition of *PXN-AS1* lead to a shortened G1 phase in K562 IR^sh-*PXN-AS1*^ and LAMA84 IR^sh-*PXN-AS1*^ cells (Fig. S9e, f). The relative mRNA levels of mTOR, Cyclin D1, CDK4, and CDK6 were also decreased in sh-*PXN-AS1*-transfected CML IR cells (Fig. 5c–f and Fig. S10c–f). Similarly, the expression levels of mTOR, Cyclin D1, CDK4, and CDK6 were also downregulated in K562 IR^sh-*PXN-AS1*^ and LAMA84 IR^sh-*PXN-AS1*^ cells (Fig. 5g and Fig. S10g). These data demonstrated that *PXN-AS1* knockdown could restrain the growth of CML IR cells and rescue the cell cycle disorder of CML IR cells via suppressing the Cyclin D/CDK4-CDK6 complex and mTOR signaling pathway in vitro.

The CML mouse model was established by injecting sh-*PXN-AS1*-transfected K562 IR cells into the tail vein of the mice. The Kaplan–Meier analysis revealed that *PXN-AS1* inhibition could also predict a favorable prognosis (Fig. 5h). The symptom of splenomegaly was less severe in K562 IR^sh-*PXN-AS1*^-injected mice than in K562 IR^sh-NC^-injected mice (Fig. 5i). In addition, the ratio of spleen/body weight was much lower in K562 IR^sh-*PXN-AS1*^-injected mice (Fig. 5j). According to the results of HE staining, it was observed that K562 IR^sh-*PXN-AS1*^-injected mice had more spleen metastasis CML cells than K562 IR^LV4-NC^-injected mice (Fig. S11a). The IHC staining indicated that the expression levels of GS, mTOR, Cyclin D1, CDK4, and CDK6 were all decreased in the spleen tissues of K562 IR^sh-*PXN-AS1*^-injected mice (Fig. S11b). These data demonstrated that *PXN-AS1* inhibition could retard the progression of CML by inhibiting GS expression and predict a favorable prognosis.

### *MiR-635* is decreased in the bone marrow samples of CML patientswith Imatinib resistance and CML IR cell lines

**Fig. 6:**
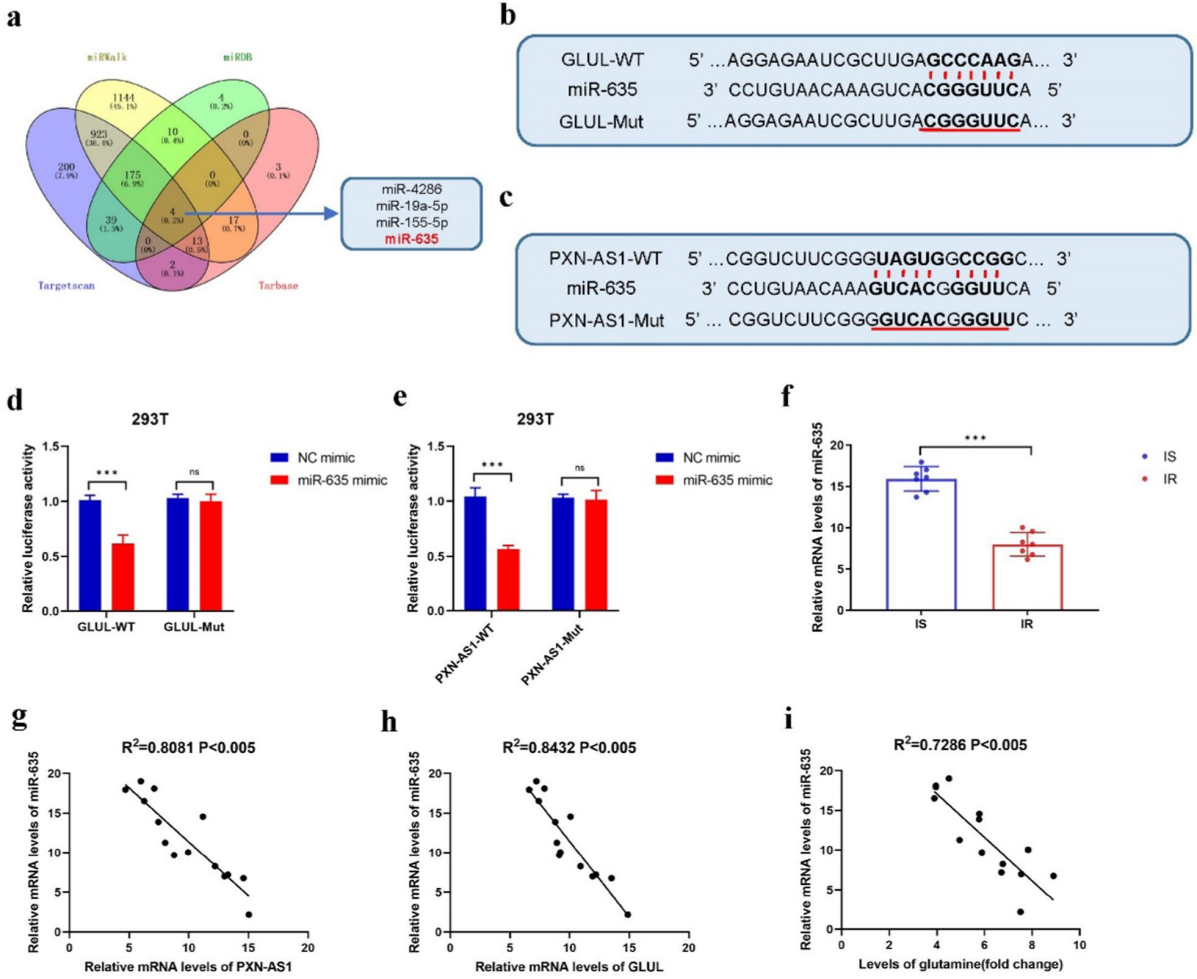
Increased level of *PXN-AS1* in bone marrow of CML patients resistant to Imatinib and CML IR cell lines. **a** Venn diagram of four RNA-RNA tools used to predict the microRNAs co-expressed with GS and *PXN-AS1* in CML. **b** The potential binding site of *miR-635* and GS. **c** The potential binding site of *miR-635* and *PXN-AS1*. **d** Dual-luciferase reporter assays between GS and *miR-635*. **e** Dual-luciferase reporter assays between *PXN-AS1* and *miR-635*. **f** Real-time PCR of relative mRNA levels of *miR-635* in bone marrow of CML patients resistant to Imatinib and those sensitive to Imatinib. **g–i** Correlation analyses between *miR-635* and GS, Gln levels and *PXN-AS1* in 7pairs of bone marrow of CML patients resistant to Imatinib and those sensitive to Imatinib. Data are expressed as mean ± standard deviation of at least three independent experiments. Statistical significance was concluded at *P < 0.05, **P < 0.01, and ***P < 0.005

To identify potential microRNAs that can bind to both *PXN-AS1* and GS, four online tools (miRWalk, miRDB, Targetscan, and Tarbase) were used for bioinformatics prediction. Consequently, four microRNAs (*miR-635*, miR-4286, miR-19a-5p, and miR-155-5p) were finally selected (Fig. 6a). According to the results of RT-PCR analysis, it was found that the levels of *miR-635* exhibited the most significant decrease in K562 IR and LAMA84 IR cells (Fig. S12a, b). It is interesting to note that *miR-635*, which was found to be downregulated in CML IR cells, has been reported to have potent anti-cancer effects. Given its potential significance, *miR-635* is indeed a promising candidate for further study in the context of CML and Imatinib resistance. The potential binding sites between *miR-635* and GS or *PXN-AS1* are shown in Fig. 6b, c. According to the results of dual-luciferase reporter assay, *miR-635* mimics reduced the luciferase activity of WT GS and WT *PXN-AS1*, but failed to influence the luciferase activity of Mut GS or Mut *PXN-AS1*, suggesting that *miR-635* probably bind to both WT GS target sites and WT *PXN-AS1* target sites (Fig. 6d, e). Further supporting its potential role in Imatinib resistance, the level of *miR-635* was found to be significantly reduced in samples from CML patients who were resistant to Imatinib compared to those who were sensitive to Imatinib, as determined by RT-PCR analysis (Fig. 6f). Additionally, *miR-635* expression exhibited a negative correlation with the levels of GS, Gln, and *PXN-AS1* in samples from CML patients who were resistant to Imatinib (Fig. 6g–i). These data demonstrated that *miR-635* was downregulated in the human bone marrow of CML patients with Imatinib resistance and CML IR cell lines, implying that *miR-635* might be an imperative factor for mediating the *PXN-AS1*/*miR-635*/GS axis.

### *PXN-AS1*/*miR-635*/GS axis is imperative for BCR::ABL1-independent Imatinib resistance in CML cells

**Fig. 7:**
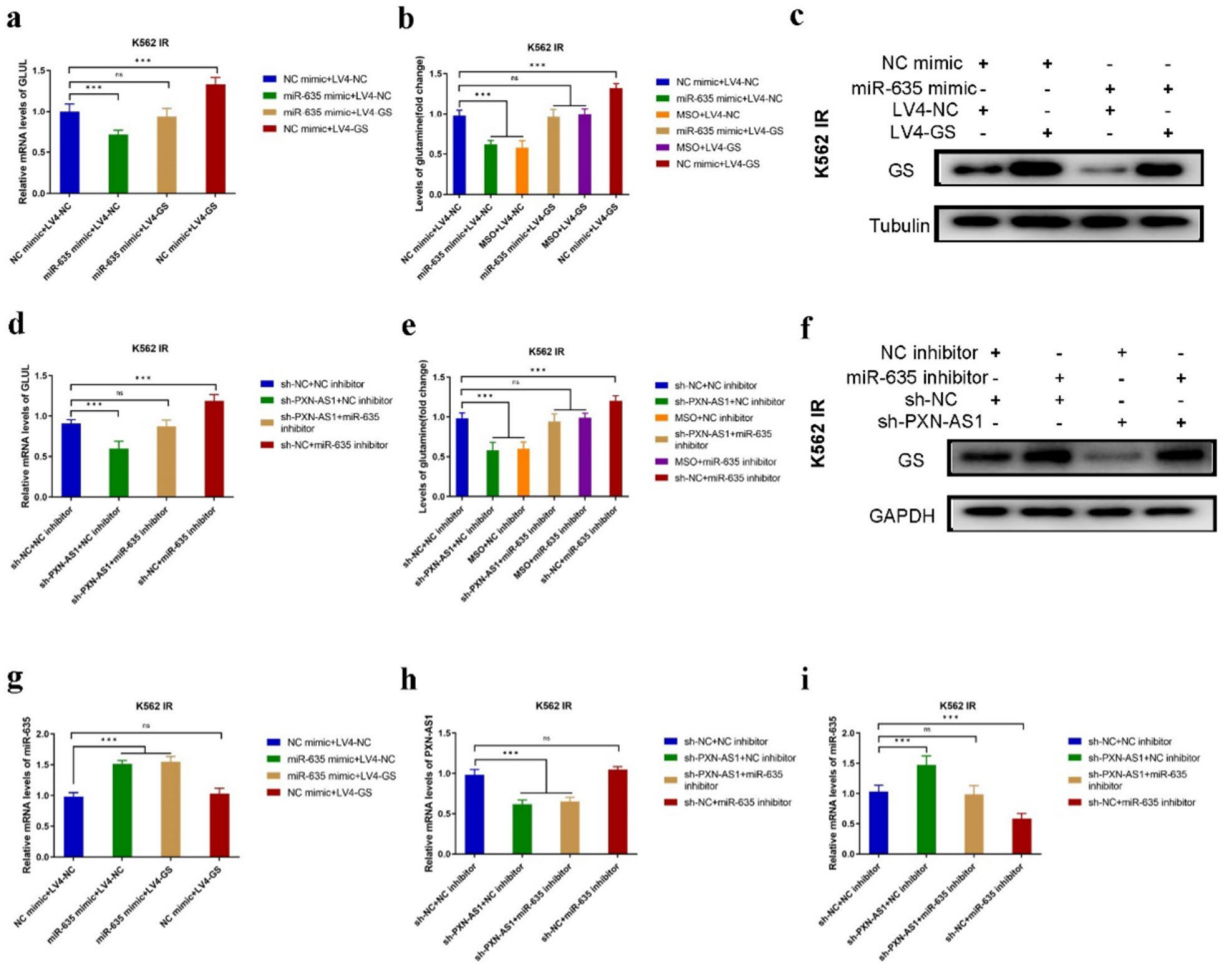
Knockdown of *PXN-AS1* mediates GS expression via upregulation of *miR-635* levels. **a** The GS mRNA levels of K562 IR cells after *miR-635* mimic transfection. **b** The Gln content of K562 IR cells after *miR-635* mimic transfection. **c** The GS expression of K562 IR cells after *miR-635* mimic transfection. **d** The GS mRNA levels of K562 IR cells after *miR-635* inhibitor transfection. **e** The Gln content of K562 IR cells after *miR-635* inhibitor transfection. **f** The GS expression of K562 IR cells after transfected with *miR-635* inhibitor. **g** The *miR-635* mRNA levels of K562 IR cells after *miR-635* mimic and LV-GS co-transfection. **h** The *PXN-AS1* mRNA levels of K562 IR cells after *miR-635* inhibitor and sh-*PXN-AS1* co-transfection. **i** The *miR-635* mRNA levels of K562 IR cells after *miR-635* inhibitor and sh-*PXN-AS1* co-transfection. Data are expressed as mean ± standard deviation of at least three independent experiments. Statistical significance was concluded at *P < 0.05, **P < 0.01, and ***P < 0.005

Next, RNA mimic and RNA inhibitor were used to explore the effect of *miR-635* on CML IR cells. The expression levels of GS were decreased in K562 IR^*miR-635*mimic^ and LAMA84 IR^*miR-635*mimic^ cells compared to K562 IR^NC mimic^ and LAMA84 IR^NC mimic^ cells, as revealed by RT-PCR and Western blotting (Fig. 7a,c,g and Fig. S13a, c, g). Moreover, the content of Gln was also decreased in K562 IR^*miR-635*mimic^ and LAMA84 IR^*miR-635*mimic^ cells (Fig. 7b and Fig. S13b). On the contrary, the expression of GS was increased in K562 IR^*miR-635*inhibitor^ and LAMA84 IR^*miR-635*inhibitor^ cells, as determined by RT-PCR and Western blotting (Fig. 7d, f and Fig. S13d, f). Similarly, the content of Gln was also increased in K562 IR^*miR-635*inhibitor^ and LAMA84 IR^*miR-635*inhibitor^ cells (Fig. 7e and Fig. S13e). In addition, *PXN-AS1* knockdown might affect the level of *miR-635* in K562 IR and LAMA84 IR cells (Fig. 7h,i and Fig. S13h, i). Therefore, we deduce that GS regulates BCR::ABL1-independent Imatinib resistance in CML cells via the *PXN-AS1*/*miR-635*/GS axis.

## Discussion

Drug resistance remains the primary factor leading to treatment failure among patients with CML. Several common causes of drug resistance in CML have been identified, but the specific molecular mechanism of drug resistance in CML is still unclear. This study aims to contribute novel ideas and approaches to address BCR::ABL1-independent drug resistance in the context of CML.

In CML patients, serum concentration after imatinib mesylate uptake is known to be approximately 5 μM [33]. In literature, there are publications involving cell lines that developed resistance to dosages comparable to those observed in patients [34]. It has not been clearly explained why the drug resistance model was applied at a low dosage as 0.1 and 0.2 μM in cell lines. Moreover, the absence of a mutation scan was seen as a shortcoming in order to speak of BCR::ABL1-independent resistance. But when the data are assessed together, the results are convincing. Although shortcoming remains, it is still the mainstream way to generate drug resistance cell lines for deep study.

Numerous studies have reported that GS and its metabolite Gln are involved in the regulation of the occurrence, invasion and metastasis, immune escape, drug resistance and other processes of a variety of malignant tumors [35–37]. Our previous study found that GS can promote the resistance of colon cancer cells to 5-fluorouracil [21]. Here we speculated that the abnormal increase of GS level might be the main cause of BCR::ABL1-independent resistance to TKIs in CML cells. Recent studies have reported that GS can promote the malignant phenotype of hepatocellular carcinoma by activating mTOR signaling pathway [20]. We also found that GS could activate the mTOR signaling pathway, which is a crucial factor for cell cycle-associated signaling pathway activation. The results of our study, on the basis of previous reports, further revealed the molecular mechanism of BCR::ABL1-independent resistance to TKIs in CML cells [13]. A large number of studies are needed to further evaluate whether GS could be a potential therapeutic target against BCR::ABL1-independent resistance.

Although unable to encode amino acids, lncRNA is powerful transcriptional regulators [38, 39]. So lncRNA plays a very important role in the process of various diseases, especially malignant tumors [40–42]. Some lncRNAs have even been used as diagnostic indicators and therapeutic targets for some malignant tumors in clinical diagnosis and treatment [43]. LncRNA can act as either a promoter or an inhibitor in hematological malignancies [44]. LncRNA *HOTTIP* was found to promote the self-renewal ability of AML stem cells, leading to the development of AML [45, 46]. Another lncRNA, called *NEAT1*, is considered to inhibit the self-renewal ability of AML stem cells by inhibiting the Wnt signaling pathway [47]. Also, *NEAT1* acts as the downstream regulator of c-Myc and participates in the inhibition of apoptosis in CML [48].

*PXN-AS1* is also a lncRNA closely related to malignant tumors. Previous studies have shown that *PXN-AS1* is involved in the regulation of the occurrence, metastasis and drug resistance of malignant tumors such as liver cancer, nasopharyngeal carcinoma, non-small cell lung cancer and malignant glioma [24–29]. In our study, *PXN-AS1* levels were significantly increased in bone marrow mononuclear cells from TKIs-resistant CML patients and in imatinib-resistant CML cell lines, and *PXN-AS1* inhibition was proved to alleviate TKIs resistance of CML IR cells. However, due to the powerful and complex functions of lncRNA and the large number of regulatory networks, only a small number of lncRNA have been approved by the FDA for clinical trials. Therefore, whether *PXN-AS1* could be chosen as a therapeutic target to combat BCR::ABL1-independent resistance to TKIs in CML remains to be a long way to go.

MiRNA is a class of endogenous small RNA molecules with transcriptional regulation function. Different from lncRNA, miRNA mainly plays the role of inhibiting gene transcription and inhibiting mRNA stability [49]. In the human body, miRNA often cooperates with lncRNA and mRNA forming a regulatory axis, which participates in the regulation of the malignant phenotype of tumor cells. For example, in lung cancer, *JPX/miR-33a-5p/Twist1* regulatory axis promotes the occurrence and metastasis of lung cancer by activating Wnt/β-catenin signaling pathway [30]. In colon cancer, *SNHG1/miR-154-5p/EZH2* regulatory axis promotes the growth of colon cancer cells by regulating cell cycle-related signaling pathways [31]. Notably, the lncRNA/miRNA/mRNA regulatory axis can also participate in the regulation of drug resistance in malignant tumors. One study found that *PVT1/miR-619-5p/Pygo2* and *PVT1/miR-619-5p/ATG14* regulatory axes could mediate gemcitabine resistance in pancreatic cancer cells by activating Wnt/β-catenin signaling pathway and autophagy signaling pathway respectively [32].

Recent studies have reported that *miR-635* has shown strong tumor suppressor activity in tumor cells of gastric cancer, nasopharyngeal carcinoma and non-small cell lung cancer, respectively [50–52]. In this study, we found that *miR-635* has an important role in inhibiting GS expression. Furthermore, the GS overexpression induced by inhibition of *miR-635* could be rescued by knockdown of *PXN-AS1*. Taken together, the *PXN-AS1/miR-635*/GS regulatory axis might serve as a potential therapeutic target against BCR::ABL1-independent resistance to TKIs in CML cells.

## Conclusions

In summary, our study revealed important findings regarding the role of lncRNA *PXN-AS1* in mediating BCR::ABL1-independent resistance to Imatinib in CML. We discovered that *PXN-AS1* activates the mTOR signaling pathway and disrupts the Cyclin D/CDK4-CDK6 complex through its interaction with GS. *PXN-AS1* achieves this by competitively binding to *miR-635*, thus regulating GS expression. Furthermore, we established that GS and its product, Gln, play a crucial role in *PXN-AS1*-mediated CML cell proliferation and cell cycle dysregulation. Our findings demonstrate that *PXN-AS1* regulates these processes through the *PXN-AS1*/*miR-635*/GS/Gln/mTOR pathway. Importantly, targeting both *PXN-AS1* and GS has shown promise as a prognostic indicator for favorable outcomes in CML. Overall, our study sheds light on how *PXN-AS1* facilitates BCR::ABL1-independent resistance to Imatinib in CML cells via the mTOR signaling pathway, mediated by GS. These findings provide valuable insights into potential new detection methods for drug resistance pathways in clinical CML treatment and identify novel targets for therapeutic interventions.

### Supplementary Information

Supplementary Material 1.

Supplementary Material 2.

Supplementary Material 3.

Supplementary Material 4.

Supplementary Material 5.

Supplementary Material 6.

Supplementary Material 7.

Supplementary Material 8.

Supplementary Material 9.

Supplementary Material 10.

Supplementary Material 11.

Supplementary Material 12.

Supplementary Material 13.

Supplementary Material 14.

## Data Availability

All data generated during this study are included in this published article [and its supplementary information files].

## Abbreviations

GS: Glutamine synthetase
CML: Chronic myeloid leukemia
Gln: Glutamine
IR: Imatinib resistant
IS: Imatinib sensitive
PXN-AS1: PXN antisense transcript 1
lncRNA: Long non‐coding RNA
RNA‐seq: RNA sequencing
PBS: Phosphate buffer saline
NC: Negative control
LV: Lentivirus
IHC: Immunohistochemistry
mTOR: Mammalian target of rapamycin
CDK: Cyclin-dependent kinase
