# Supplementary material for: Long non-coding RNA PXN-AS1 promotes glutamine synthetase-mediated chronic myeloid leukemia BCR::ABL1-independent resistance to Imatinib via cell cycle signaling pathway
Source: Cancer Cell Int. 2024 May 29;24:186. doi: 10.1186/s12935-024-03363-9 (PMC11138077; doi:10.1186/s12935-024-03363-9)
Supplement: Supplementary file 1 — Supplementary Material 1. [file 12935_2024_3363_MOESM1_ESM.docx]

Table S1: Sequences of the genes of RT-PCR used in this study

| genes | sequences |
| --- | --- |
| GLUL | F: GGCACACCTGTAAACGGATAATG |
|  | R: CTGCTCCCACACCACAGTAATAT |
| Cyclin D1 | F: CCTCGGTGTCCTACTTCAAATGT |
|  | R: TTCATCTTAGAGGCCACGAACAT |
| CDK4 | F: GAGCATGTAGACCAGGACCTAAG |
|  | R: GTTCCACCACTTGTCACCAGAAT |
| CDK6 | F: TGACCAGCAGCGGACAAAT |
|  | R: GGACTGGAGCAAGACTTCGG |
| PCNA | F:GGCTCCATCCTCAAGAAGGTGTTG |
|  | R:GCCAAGGTATCCGCGTTATCTTCG |
| mTOR | F:GCAGAGACTTGATGGAGGAGAAA |
|  | R: ACACAGCTTAGGACATGGTTCAT |
| PXN-AS1 | F: TTCACGAATGGCGTGAAGCA |
|  | R: TGGGAAACGCCTAGCAAGTA |
| β-Actin | F: CCTTCCTGGGCATGGAGTC |
|  | R: TGATCTTCATTGTGCTGGGTG |

Table S2: Sequences of the miRNAs of RT-PCR used in this study

| miRNAs | sequences |
| --- | --- |
| miR-635 | ACTTGGGCACTGAAACAATGTC |
| miR-4286 | ACCCCACUCCUGGUACC |
| miR-19a-5p | AGUUUUGCAUAGUUGCACUACA |
| miR-155-5p | UUAAUGCUAAUCGUGAUAGGGGUU |

Table S3: Sequences of the shRNA used in this study

| shRNA | sequences |
| --- | --- |
| sh-GLUL-1 | TTCGATGGCTCTAGTACTTTA |
| sh-GLUL-2 | CCAGGAGAAGAAGGGTTACTT |
| sh-GLUL-3 | ATAACCACTGCTTCCATTTAA |
| sh-NC | CACACCTGTAAACGGATAATG |
| sh-PXN-AS1-1 | GCAAAGAGGAAGCACGCAAAT |
| sh- PXN-AS1-2 | GGCACTGATGTGAGCACATCA |
| sh- PXN-AS1-3 | GGAGTGACTCCTACTTGCTAG |
| sh-NC | GTGGTAGTGCACCTCACAGTA |

Table S4: Sequences of the RNA mimic used in this study

| RNA mimic | sequences |
| --- | --- |
| miR-635 mimic | F:ACUUGGGCACUGAAACAAUGUCC |
|  | R:ACAUUGUUUCAGUGCCCAAGUUU |
| NC mimic | F: UUGUACUACACAAAAGUACUG |
|  | R: GUACUUUUGUGUAGUACAAUU |

Table S5: Sequences of the RNA inhibitor used in this study

| RNA inhibitor | sequences |
| --- | --- |
| miR-635 inhibitor | GGACAUUGUUUCAGUGCCCAAGU |
| NC inhibitor | UCUACUCUUUCUAGGAGGUUGUGA |

Table S6: Information of online tools used in this study

| bioinformatics tools | websites |
| --- | --- |
| miRWalk | http://mirwalk.umm.uni-heidelberg.de/ |
| miRDB | https://mirdb.org/ |
| Targetscan | http://www.targetscan.org/ |
| Targetbase | http://mirtarbase.mbc.nctu.edu.tw/index.html |

Table S7. Relationship between GS Expression and Clinicopathologic Features of CML Patients(n=14)

| Features | Relative GS expression levels | | *P* Value |
| --- | --- | --- | --- |
|  | High (n=7) | Low (n=7) |  |
| Age |  |  |  |
| ≤50 | 3 | 2 | 0.577 |
| >50 | 4 | 5 |  |
| Gender |  |  |  |
| Male | 4 | 3 | 0.447 |
| Female | 3 | 4 |  |
| Imatinib resistance |  |  |  |
| Yes | 6 | 2 | 0.031 |
| No | 1 | 5 |  |
