# Supplementary figures and images for: Long non-coding RNA PXN-AS1 promotes glutamine synthetase-mediated chronic myeloid leukemia BCR::ABL1-independent resistance to Imatinib via cell cycle signaling pathway
Source: Cancer Cell Int. 2024 May 29;24:186. doi: 10.1186/s12935-024-03363-9 (PMC11138077; doi:10.1186/s12935-024-03363-9)

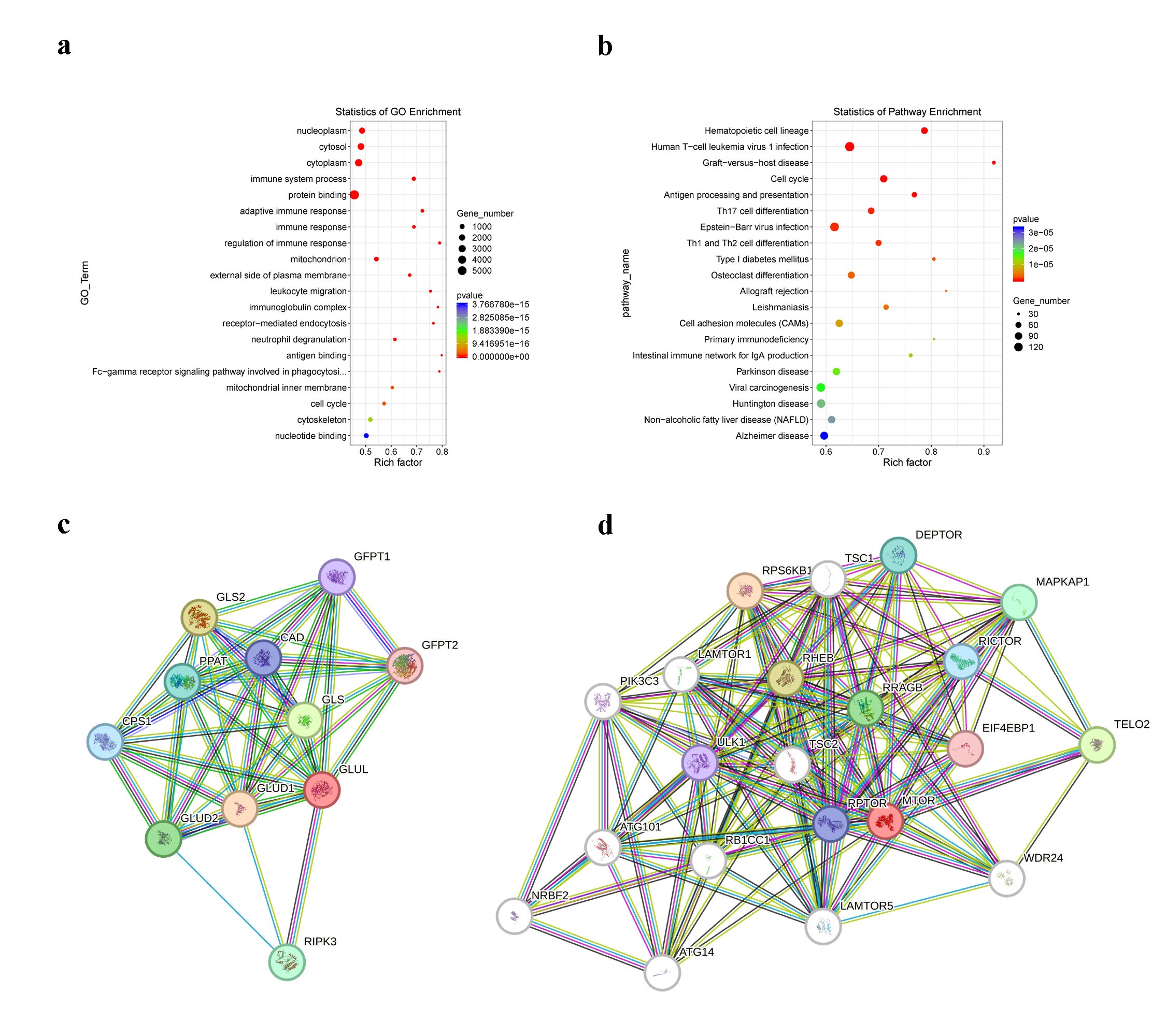

Supplement: Supplementary file 2 — Supplementary Material 2. [file 12935_2024_3363_MOESM2_ESM.jpg]

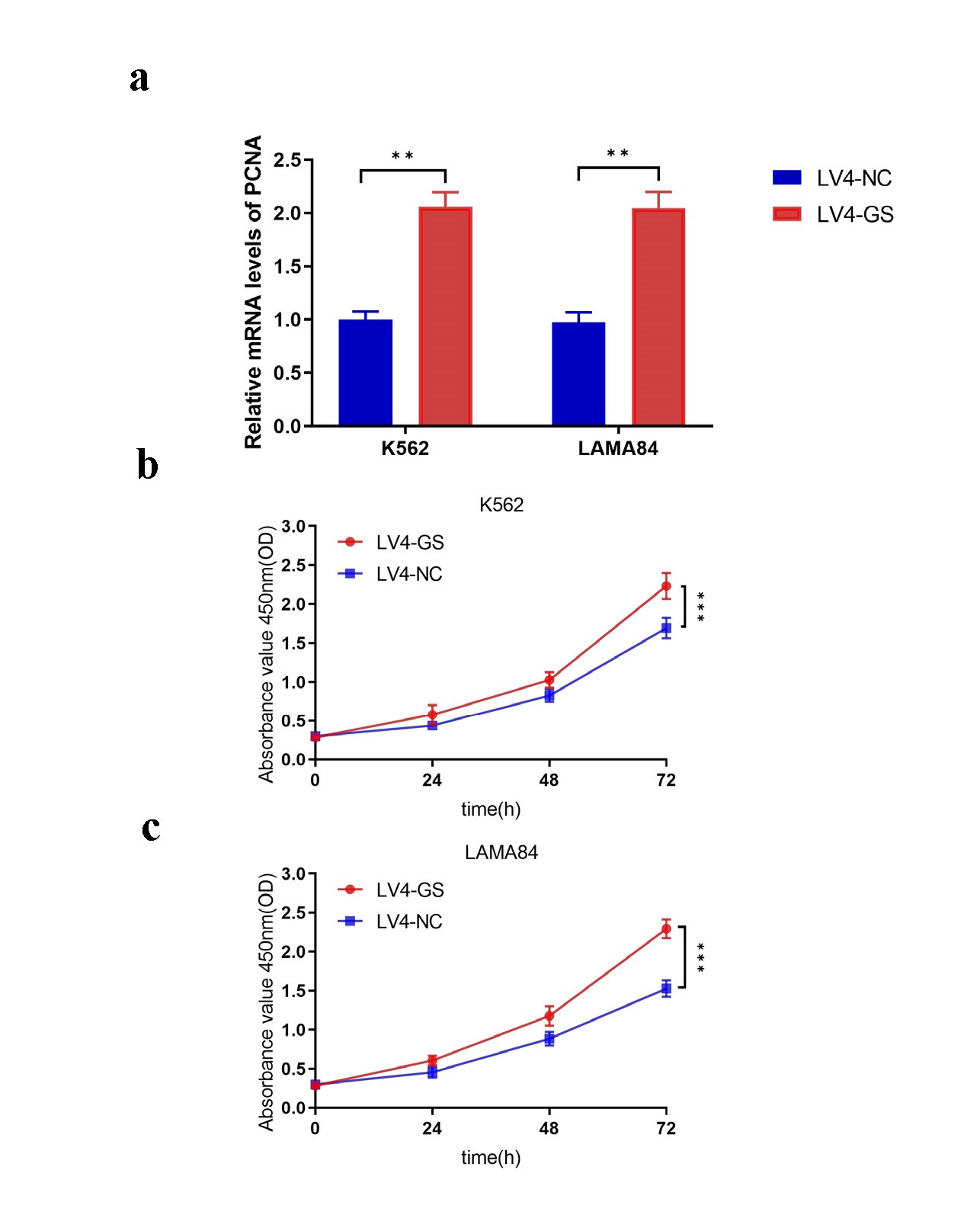

Supplement: Supplementary file 3 — Supplementary Material 3. [file 12935_2024_3363_MOESM3_ESM.jpg]

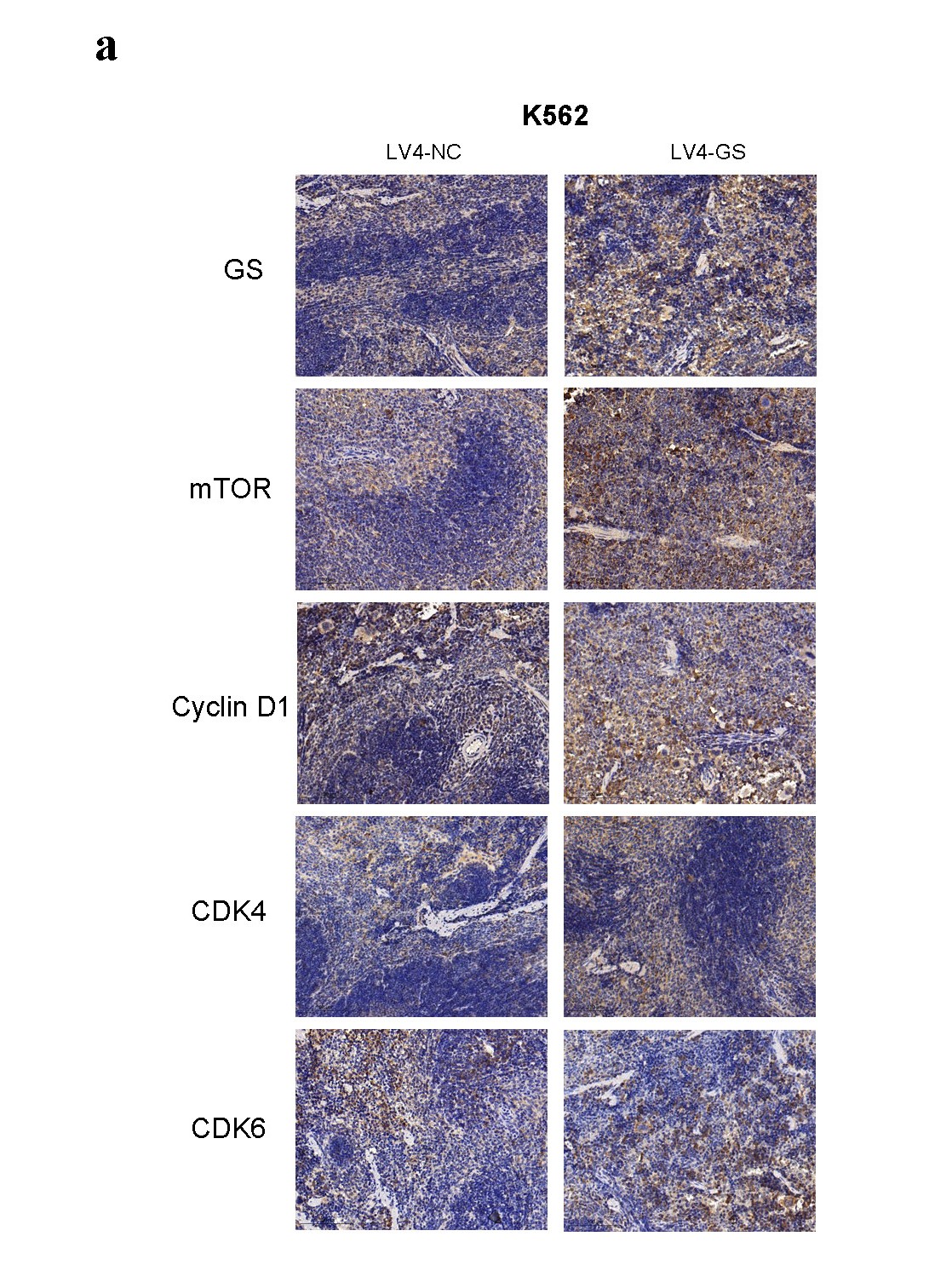

Supplement: Supplementary file 4 — Supplementary Material 4. [file 12935_2024_3363_MOESM4_ESM.jpg]

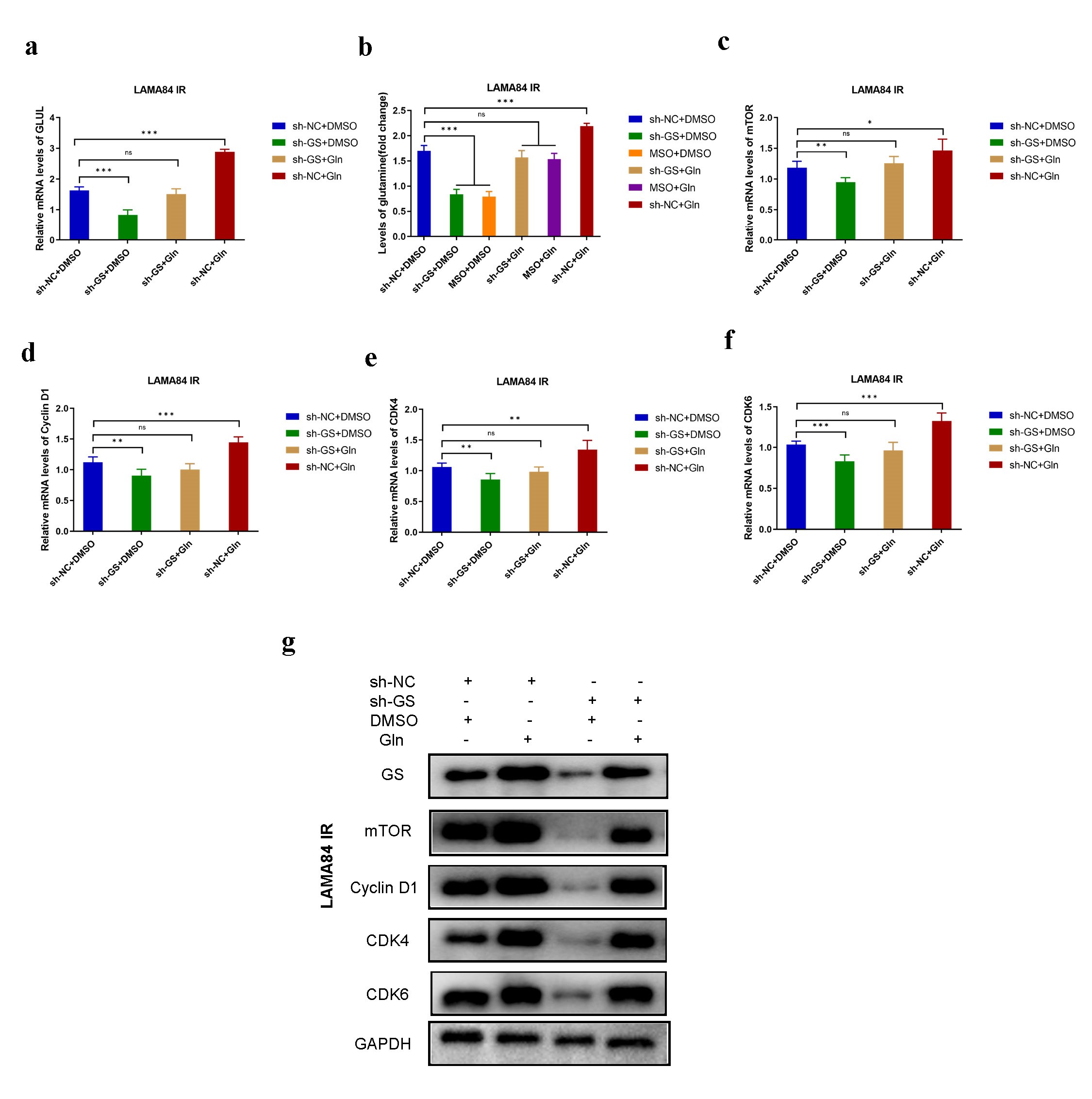

Supplement: Supplementary file 5 — Supplementary Material 5. [file 12935_2024_3363_MOESM5_ESM.jpg]

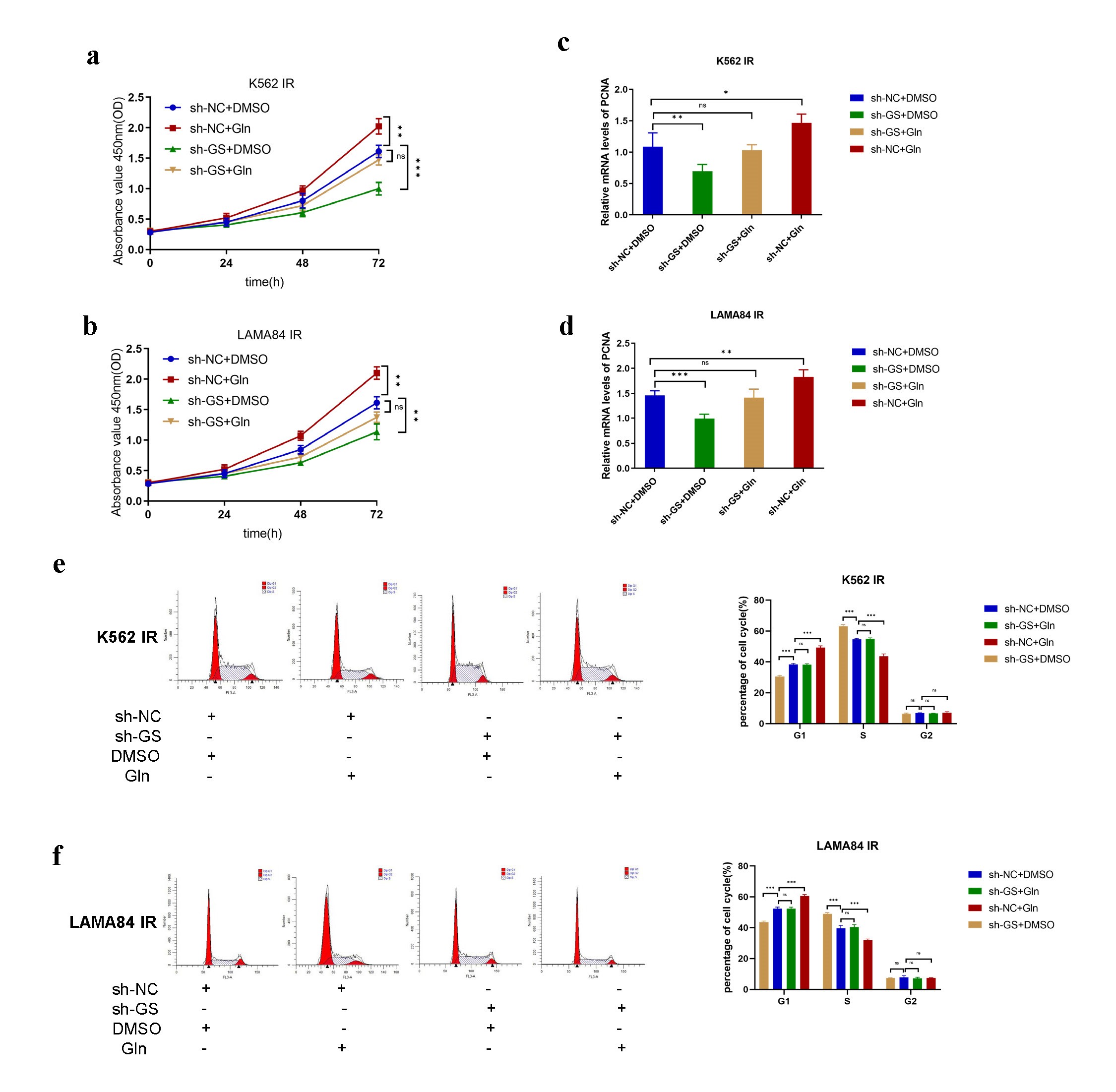

Supplement: Supplementary file 6 — Supplementary Material 6. [file 12935_2024_3363_MOESM6_ESM.jpg]

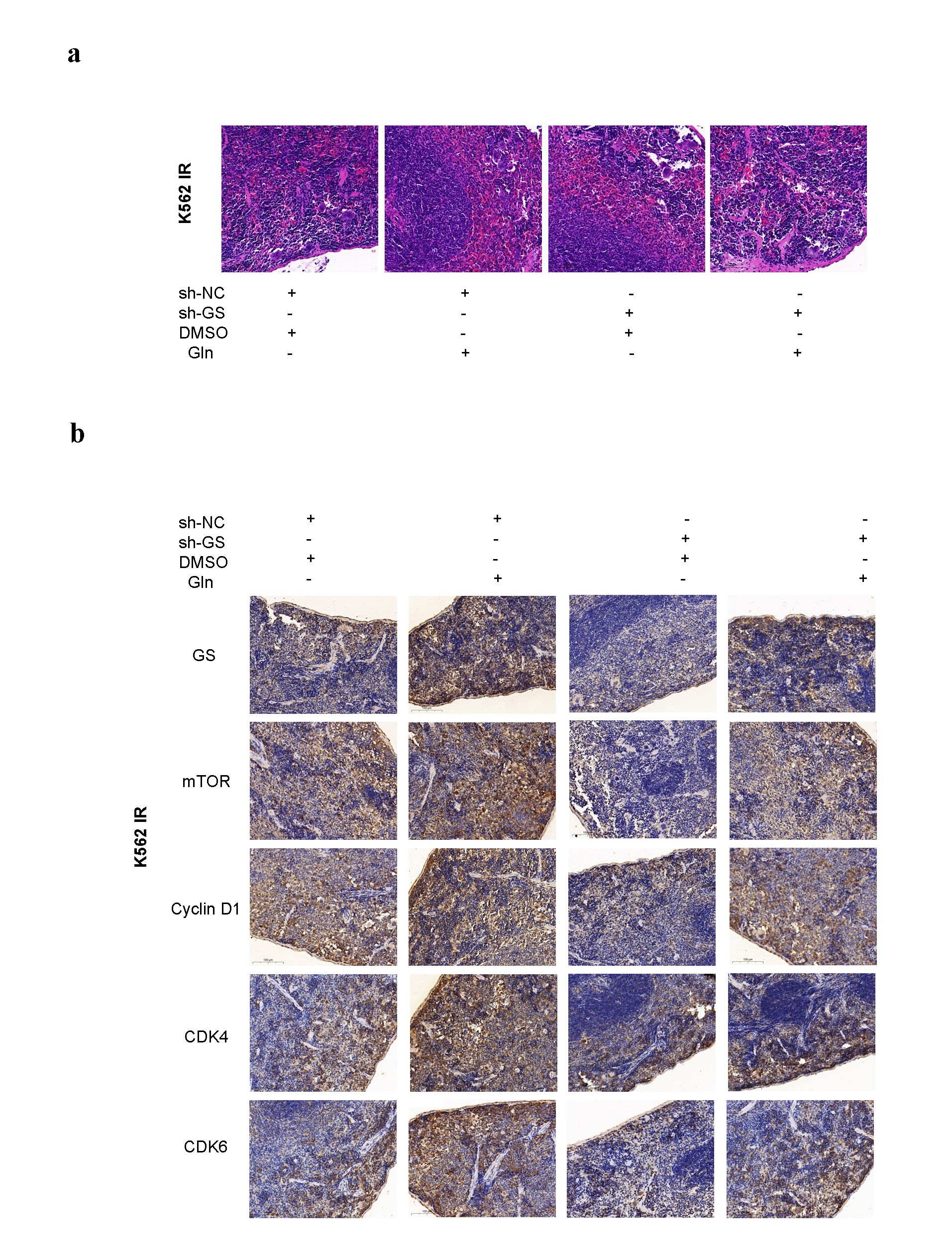

Supplement: Supplementary file 7 — Supplementary Material 7. [file 12935_2024_3363_MOESM7_ESM.jpg]

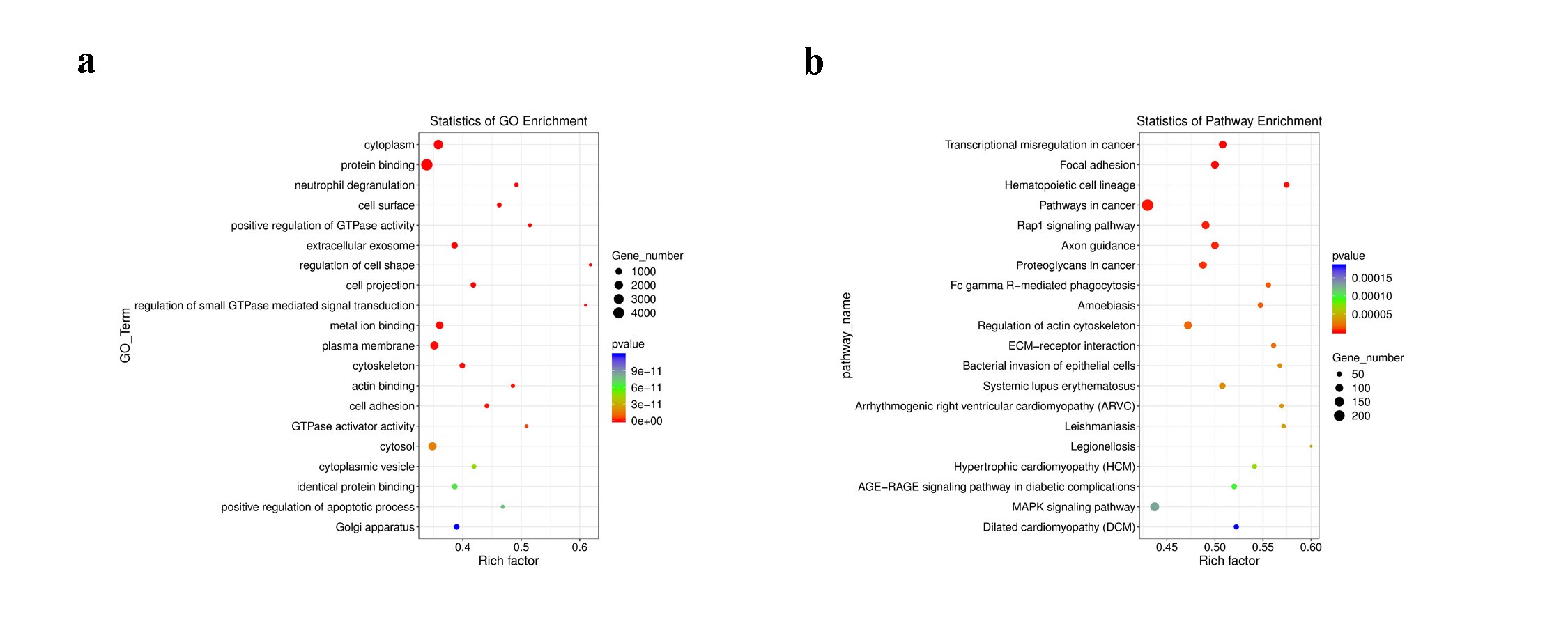

Supplement: Supplementary file 8 — Supplementary Material 8. [file 12935_2024_3363_MOESM8_ESM.jpg]

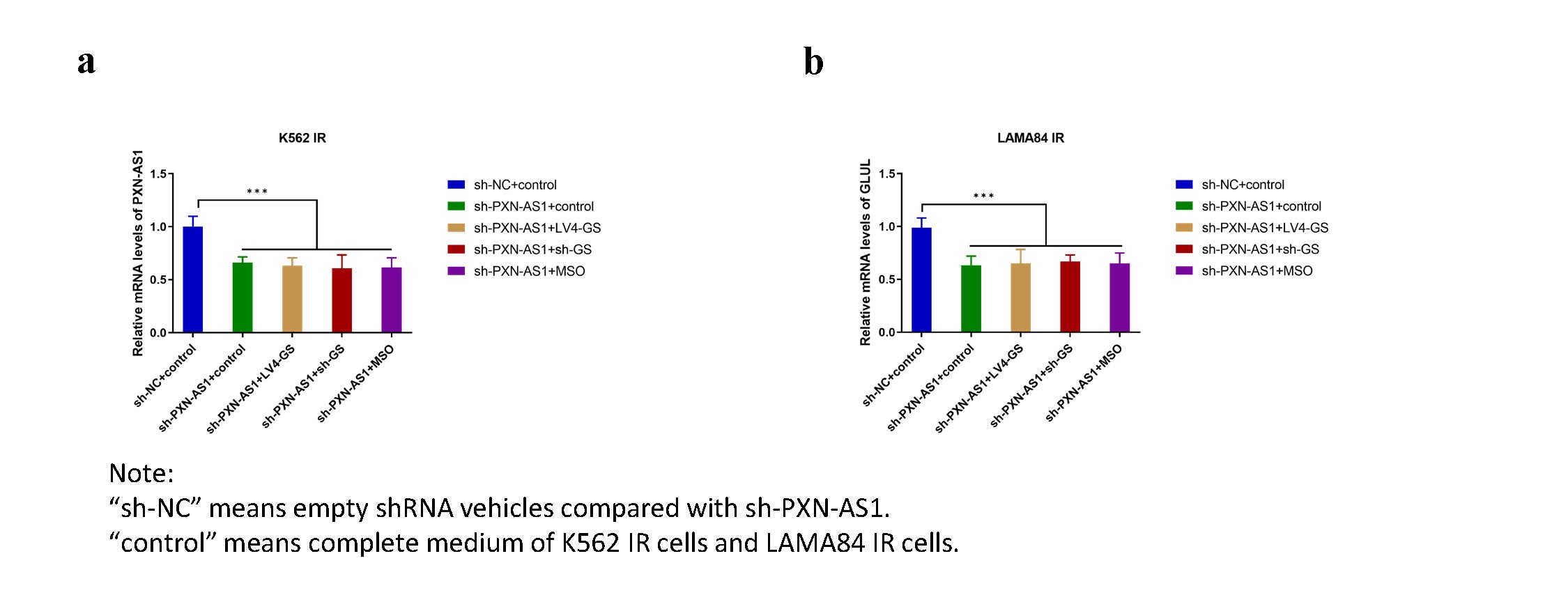

Supplement: Supplementary file 9 — Supplementary Material 9. [file 12935_2024_3363_MOESM9_ESM.jpg]

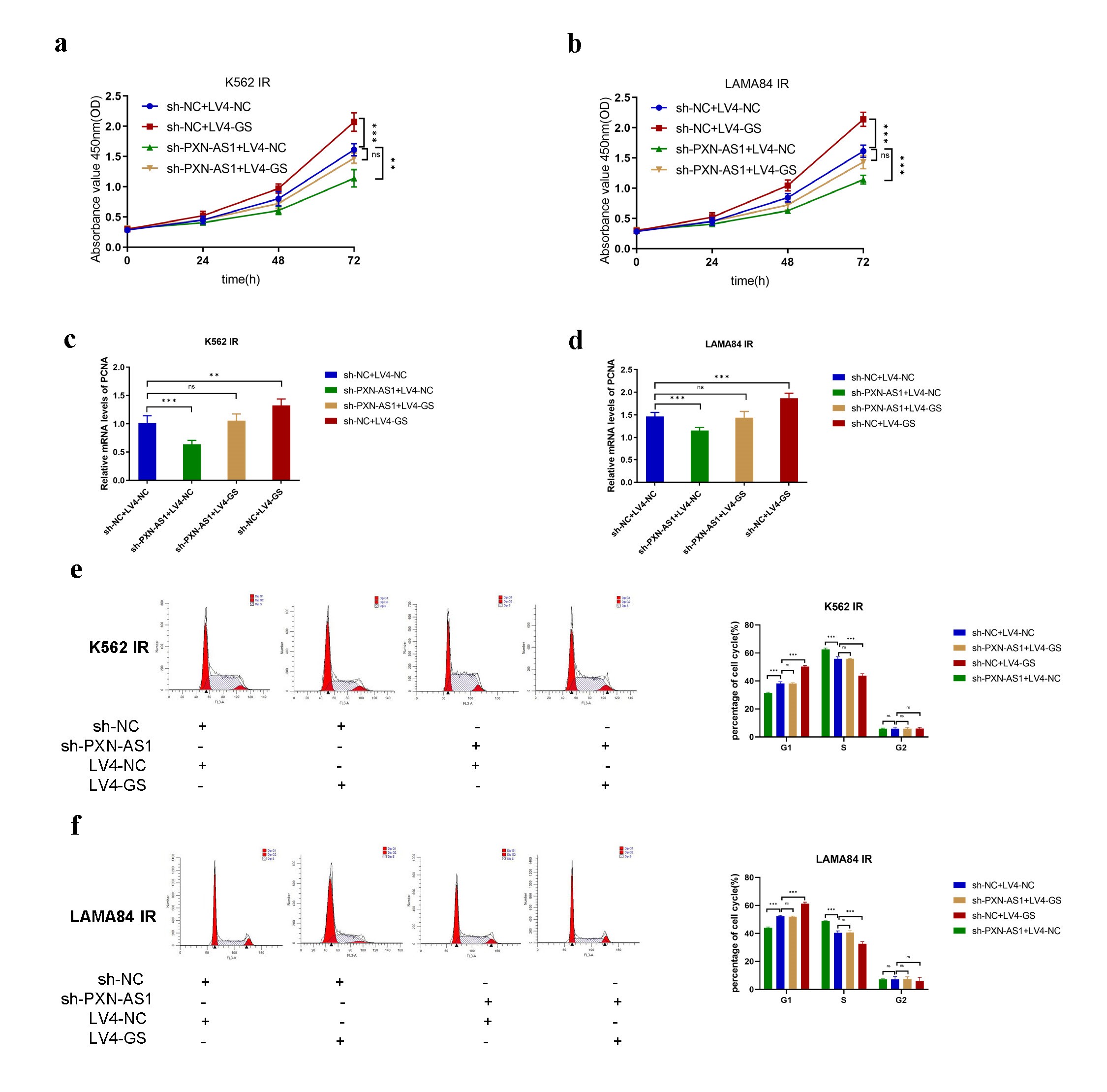

Supplement: Supplementary file 10 — Supplementary Material 10. [file 12935_2024_3363_MOESM10_ESM.jpg]

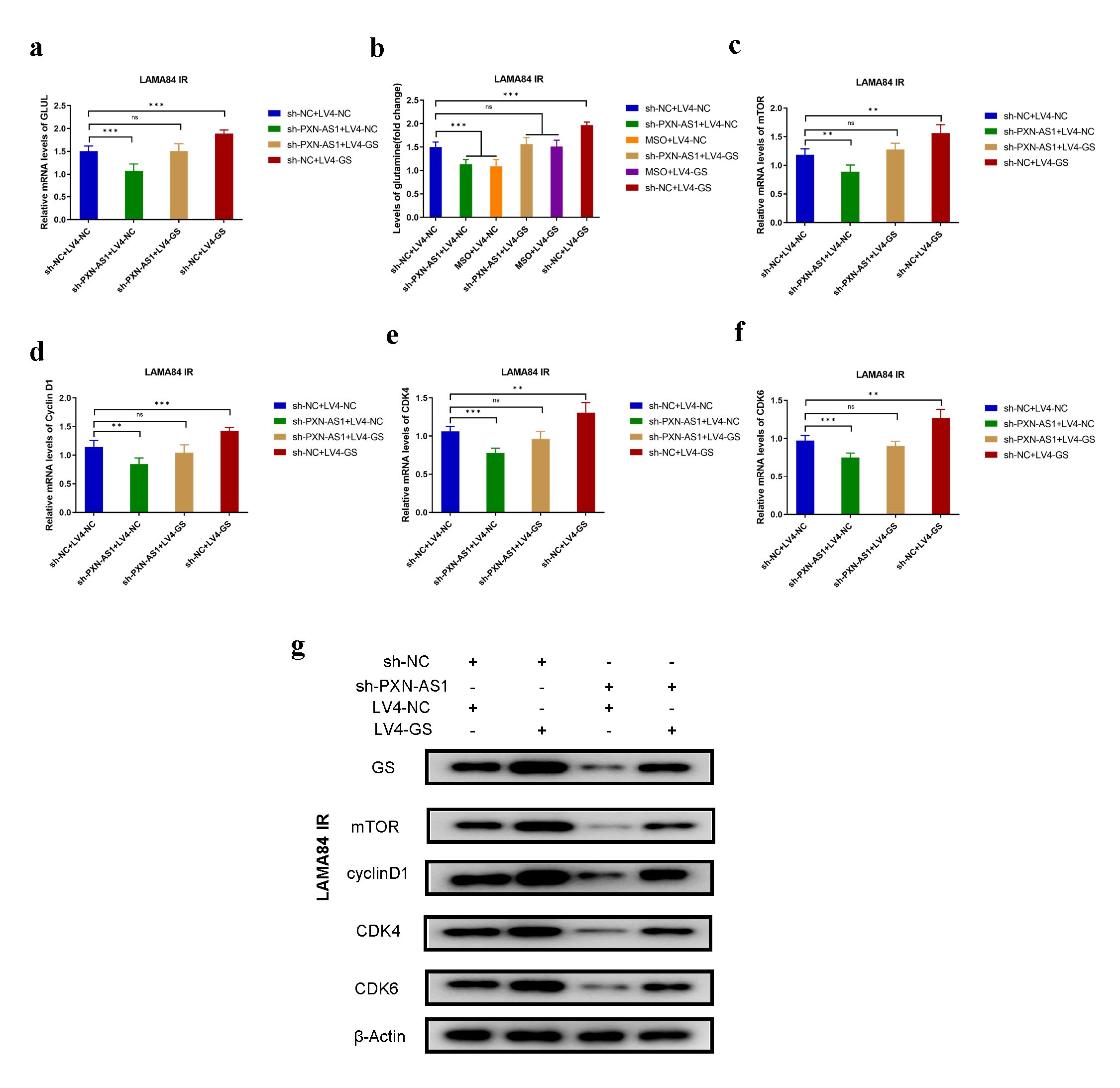

Supplement: Supplementary file 11 — Supplementary Material 11. [file 12935_2024_3363_MOESM11_ESM.jpg]

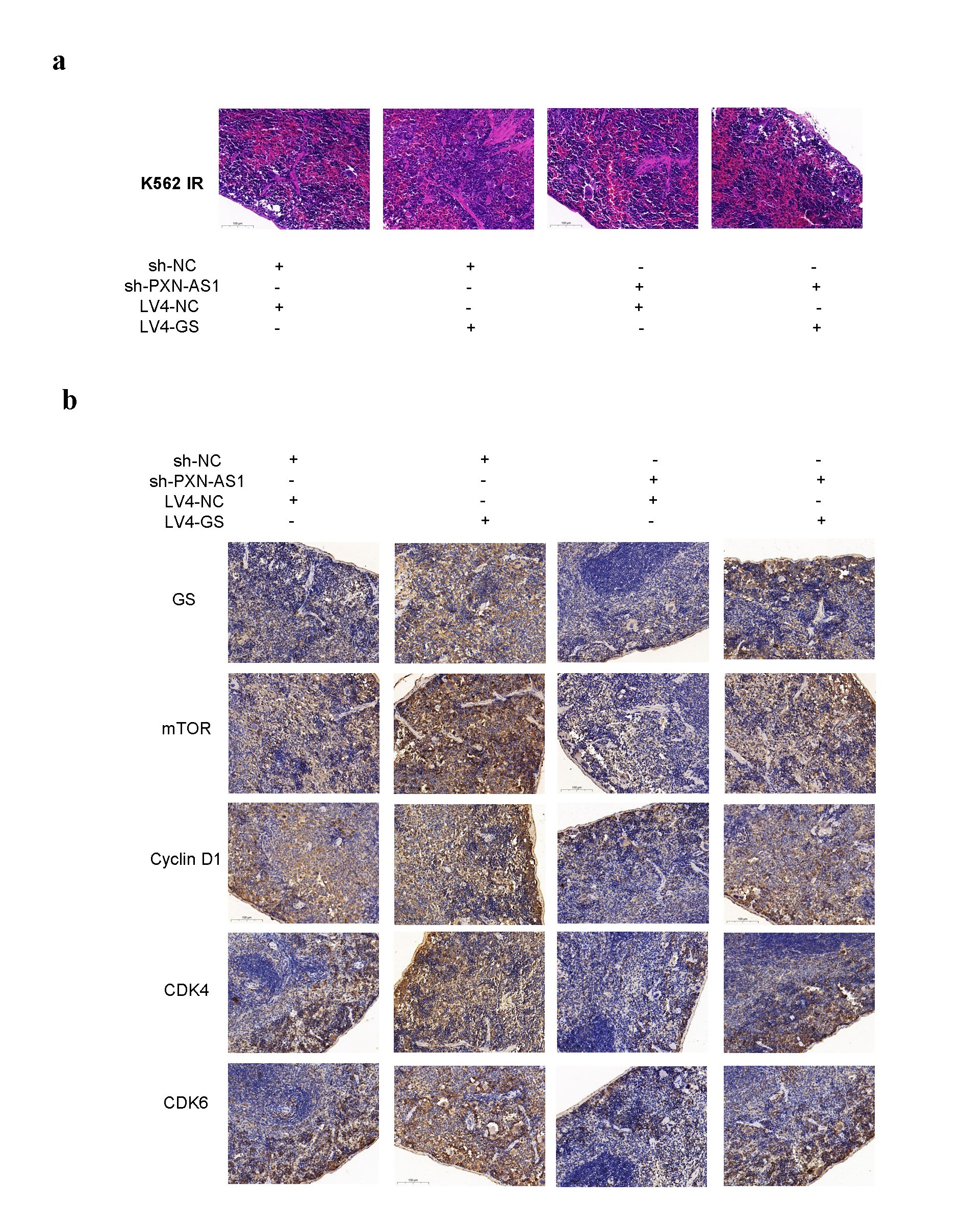

Supplement: Supplementary file 12 — Supplementary Material 12. [file 12935_2024_3363_MOESM12_ESM.jpg]

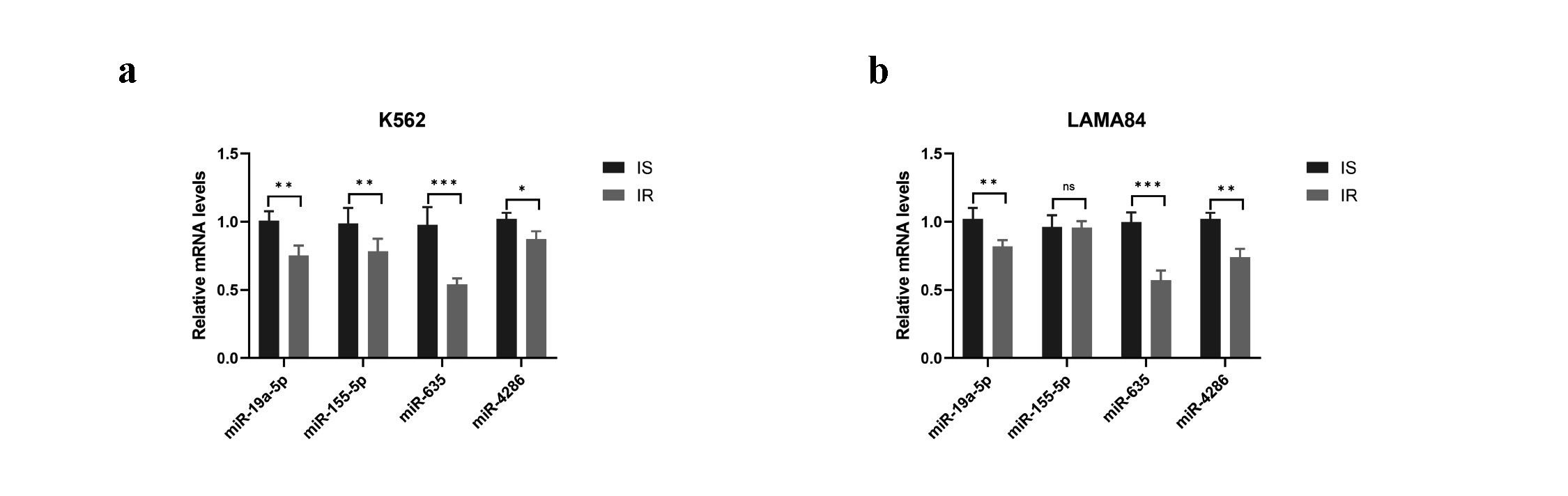

Supplement: Supplementary file 13 — Supplementary Material 13. [file 12935_2024_3363_MOESM13_ESM.jpg]

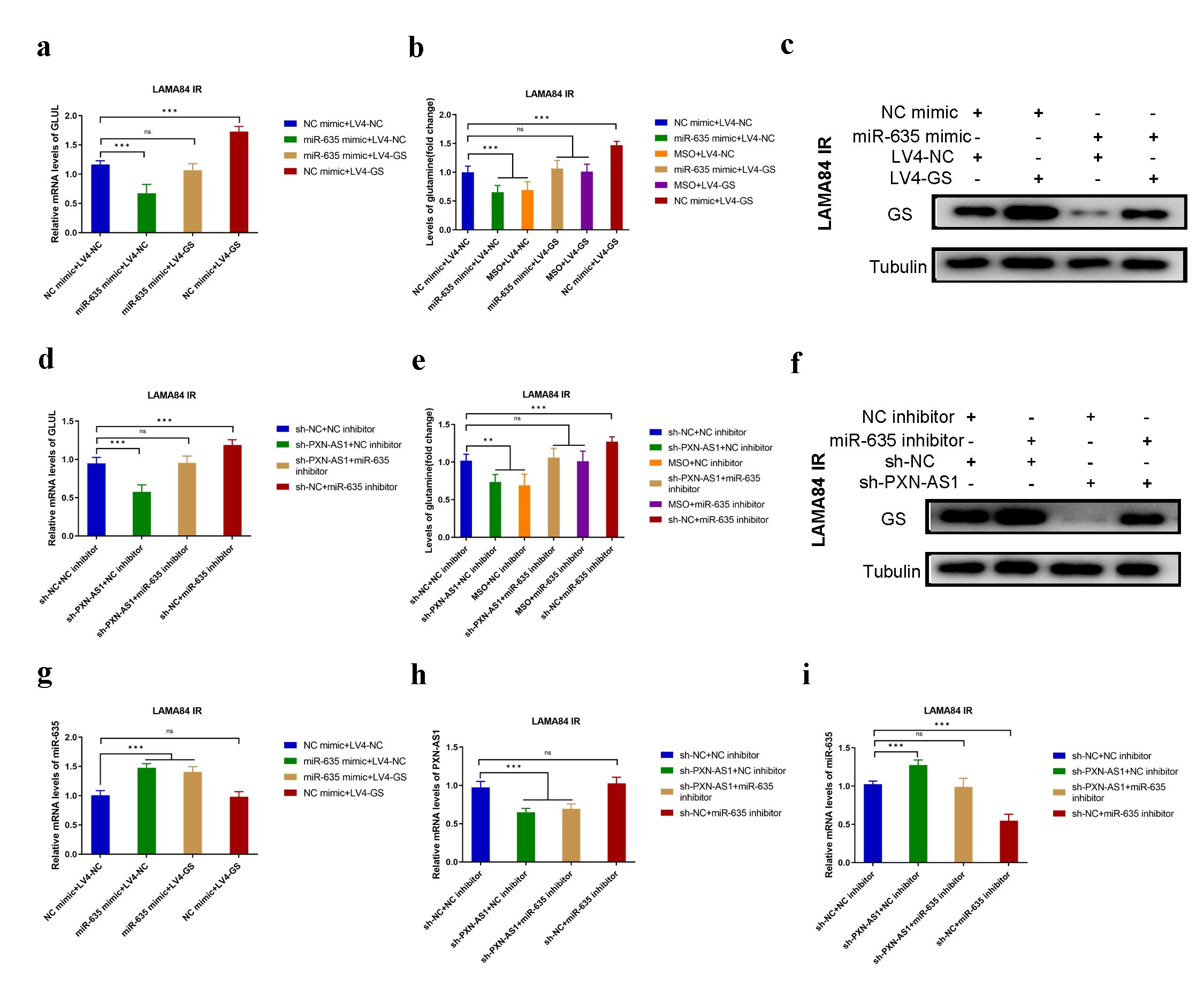

Supplement: Supplementary file 14 — Supplementary Material 14. [file 12935_2024_3363_MOESM14_ESM.jpg]
